# Real-world use of an etanercept biosimilar including selective *versus* automatic substitution in inflammatory arthritis patients: a UK-based electronic health records study

**DOI:** 10.1093/rap/rkac056

**Published:** 2022-07-27

**Authors:** Roxanne Cooksey, Sinead Brophy, Jonathan Kennedy, Michael Seaborne, Ernest Choy

**Affiliations:** CREATE Centre, Section of Rheumatology, Division of Infection and Immunity, School of Medicine, Cardiff University, Cardiff; Health Data Research UK, School of Medicine, Swansea University, Swansea, UK; CREATE Centre, Section of Rheumatology, Division of Infection and Immunity, School of Medicine, Cardiff University, Cardiff; Health Data Research UK, School of Medicine, Swansea University, Swansea, UK; CREATE Centre, Section of Rheumatology, Division of Infection and Immunity, School of Medicine, Cardiff University, Cardiff

**Keywords:** RA, PsA, AS, biosimilars, etanercept

## Abstract

**Objective:**

Biosimilars are approved as an alternative treatment to their originators. We compared the clinical outcomes of etanercept (ETN) biosimilar compared with ETN originator in real-world practice, from two local health boards in Wales with different policies on switching: automatic *vs* selective.

**Methods:**

Data from the Secure Anonymised Information Linkage (SAIL) databank in Wales were used to create a retrospective cohort study using linked primary and secondary care data. Patients aged ≥18 years with diagnosis codes for RA, PsA or AS were included. Outcomes included treatment failure and DAS-28 score (for RA). The local health board with a policy of automatic switching (i.e. clinician/nurse involvement not mandated) is labelled as automatic switch area, and the other, which required clinician/nurse supervision, as selective switch.

**Results:**

Of 8925 individuals with inflammatory arthritis, 13.3% (365) received ETN biosimilar and 31.5% (863) ETN originator. The treatment discontinuation rate was similar for ETN biosimilar and originator by Kaplan–Meier analysis. More biosimilar failure patients were treated in the automatic switch area (15 *vs* 4.8%). In the automatic switch area, 28.8% (75 of 260) of patients switched automatically from ETN originator to biosimilar compared with 10.5% (11 of 105) in the selective switch area. ETN biosimilar reduced DAS-28 by 1.6 ± 1.8 in the selective switch area *vs* 0.4 ± 0.6 in the automatic switch area.

**Conclusion:**

The ETN biosimilar was well tolerated. Fewer people were switched using selective policy, but this was associated with lower failure rates. Automatic switch policy led to more patients being switched and did not lead to significant worsening of disease.

Key messages

- ETN biosimilar is well tolerated in individuals with inflammatory arthritis.
- Selective switching from ETN originator to ETN biosimilar was associated with lower failure rates.
- The difference in disease activity pre- and post-ETN biosimilar was largest when selectively switched.

## Introduction

The biologic DMARDs (bDMARDs) have revolutionized treatment of inflammatory conditions, including inflammatory arthritic conditions, RA, PsA and AS. National and international guidelines recommend the use of bDMARDs to treat patients with moderate to severe inflammatory arthritis who do not respond well to conventional synthetic DMARDs (csDMARDs) [1–5]. The etanercept (ETN) originator is a TNF antagonist that has been recommended to treat inflammatory arthritis in the UK for many years [6–8]. Following the expiry of its patent, various biosimilars have been developed, including SB4, which has been licensed to treat inflammatory arthritis in the UK [9]. Clinical trial data on RA patients have demonstrated that the ETN biosimilar has equivalent efficacy and a comparable safety profile to the originator product [10–12].

Population-based cohort studies and systematic reviews have also demonstrated that the ETN biosimilar is similar to its originator in terms of efficacy [13–18] and safety [15, 18–20] in the treatment of inflammatory arthritis. No differences in efficacy or safety have been reported in ETN biosimilar-treated patients with previous biologics or ETN originator compared with biologic-naïve patients [17, 21].

Yet despite these findings, switching from an originator to biosimilar product remains a controversial topic. Concerns regarding their safety and efficacy have been raised [22], and both rheumatologists and patients have expressed their concerns regarding the potential differences between originator and biosimilar products [23].

The ETN biosimilar costs 10–50% less than the ETN originator [9, 24, 25]. However, authors highlight the need to balance the cost saving from switching to the cost of an unsuccessful switching process, such as sick leave, adverse events, rheumatology appointments and unused biosimilar products [26]. Although some studies report a high level of continued treatment with ETN biosimilars, the nocebo effect [27] has been observed, whereby subjective factors, such as self-reported measures, are associated with ETN biosimilar discontinuation [14, 15, 26, 28].

Supportive communication and education have been highlighted as important factors to achieve high rates of switching [28, 29]. In one study, 99% of patients agreed to switch to the ETN biosimilar after an education programme comprising a face-to-face discussion with a rheumatologist, written information and a patient information leaflet. These patients were also provided with a helpline telephone number to call for further information or to report adverse events. Subsequently, the rate of discontinuation owing to loss of efficacy or adverse events was low [29].

A single-centre clinic of a French hospital has also reported high acceptance rates of a non-mandatory switching from the ETN originator product to a biosimilar (92%, 48 of 52). The main reason for acceptance was a positive opinion of a clinician [28]. Clinicians, rather than patient characteristics, have been associated with the increased likelihood of switching to an ETN biosimilar [30]. In the UK, patients have reported that improved communication from health-care professionals would increase their acceptance rate of biosimilars [31].

The majority (86%) of patients rate their experience of switching to a biosimilar as a positive one. However, 15% felt pressured to switch [28]. In the UK, a study of non-medical switching, managed at a dedicated clinic, found that 43% of patients regarded the experience as positive, whereas 23% did not; the remainder were indifferent or failed to respond [32].

With a lack of clear guidance regarding switching from originator ETN to a biosimilar, findings regarding the effect of mandatory *vs* selective switching are less conclusive and often difficult to compare. In a non-mandatory switching environment in a UK hospital, patients were switched only if they gave consent. Of the 72 patients who consented to switch, 26.4% switched back to ETN originator. The reasons for switching back to ETN were largely loss of effect. The authors also found that the nocebo effect might have been a factor, owing to failure of the ETN biosimilar being associated with the duration of previous ETN originator, in addition to subjective measures [26]. In another study from the UK, after mandatory ETN biosimilar switching of all ETN originator patients, there was a good response, with 84% continuing with treatment and even reporting fewer injection site reactions [33].

In Wales, UK, there are seven local health boards (LHBs) that deliver health care to the nation. Each LHB area supports the population of Wales, within the range of 100 000 to 700 000 residents per LHB area. The two LHBs in this study, each serving an urban population of between 400 000 and 445 000, offer a unique opportunity to investigate the real-world use of an ETN biosimilar. One LHB area advocates the automatic use or substitution (automatic area) of the ETN originator with an ETN biosimilar, whereas the other offers selective use of substitution (selective area) with an ETN biosimilar. In the selective substitution area, use of the ETN biosimilar is at the discretion of the rheumatologist and involves discussing the options with patients involving their treatment. Both LHB areas have three rheumatology clinic locations each.

Herein, using linked, routinely collected health data stored as electronic health records available for research, we explore the use, effectiveness and health outcomes of ETN biosimilar use compared with the ETN originator, and we investigate the effect of automatic or selective ETN biosimilar use.

## Methods

A retrospective cohort of patients was created by using linked electronic health records from the Secure Anonymised Information Linkage (SAIL) databank. The SAIL databank houses multi-sourced records from 5 million of the living and deceased population of Wales, in a highly secure data repository that allows the retrospective and prospective follow-up through health and social care datasets. For example, routine data that are collected in primary care from general practitioner (GP) consultations are available in the SAIL databank, along with secondary care data, including in-patient, out-patient and accident and emergency data. Data linkage of multiple data sources can provide an exceptional level of follow-up and support longitudinal, epidemiological studies. Additional data were extracted from the Cellma dataset, an electronic health record system used by six rheumatology departments. This included biologic drugs taken, the location of the LHB and DASs, where available. The DAS-28 was included for analysis ≤1 year pre- and post-treatment, DAS-28 scores outside of a 1-year time frame, pre- or post-treatment, were not included in the DAS-28 analysis linking with the primary care dataset; data were obtained on prescribed csDMARDs and primary care encounters, whereby READ codes were present (Supplementary Table S1, available at *Rheumatology Advances in Practice*). Codelist libraries are available in Supplementary Tables S2–S4, available at *Rheumatology Advances in Practice* online.

The assumption was made that treatment failure of ETN originator and ETN biosimilar occurred when an additional biologic treatment was present in rheumatology records in place of the ETN originator or ETN biosimilar.

The records are anonymized using a split-file approach; the demographic and clinical data were divided and sent to a third party, where a unique linking field was applied, removing any identifiers. This allowed the files to be recombined later and for data to be linked across datasets.

The created cohort comprised individuals with International Classification of Diseases, Tenth Revision, Clinical Modification (ICD-10-CM) codes for RA (69896004), PsA (33339001) and AS (9631008). These were from rheumatology clinic data held in the Cellma dataset within the SAIL databank. Currently, two LHBs contribute data to Cellma. The two LHBs take different approaches to ETN biosimilar prescribing. The prescribing policy of one area is to switch ETN originator patients to ETN biosimilar automatically. At the other LHB, rheumatologists can decide selectively whether or not to use the biosimilar.

Data held in the SAIL databank are anonymized; therefore, no ethical approval is required as per the Data Protection Act 2018 [34]. All data contained in SAIL have permission from the relevant Caldicott Guardian or Data Protection Officer. This study has been approved by the SAIL databank Information Governance Review Panel.

## Results

### Cohort characteristics

The cohort comprised 8925 patients with RA, PsA and AS, who were treated at six rheumatology clinics within two LHBs in Wales, UK. Of these, 365 patients had received the ETN biosimilar; 279 were ETN naïve, and 86 switched from the originator. The characteristics of these patients are provided in Table 1.

**Table 1:** Characteristics of inflammatory arthritis patients from two rheumatology local health board areas in Wales treated with etanercept originator, etanercept biosimilar and those switched from etanercept originator to biosimilar

Parameter	**ETN originator patients (no ETN biosimilar,** ***n* = 777**	**ETN biosimilar patients (no ETN,** ***n* = 279)**	ETN biosimilar previously exposed to ETN originator (*n* = 86)
Female, % (*n*)	63.6 (494)	60.6 (169)	52.3 (45)
BMI, mean (s.d.), kg/m^2^	27.2 (5.9)	26.6 (5.6)	26.3 (5.5)
Age at diagnosis, mean (s.d.), years^c^	44.5 (13.8)	51.9 (14)	53.4 (12.0)
Diagnosis of RA, % (*n*)	68.9 (535)	70.3 (196)	58.1 (50)
Diagnosis of AS, % (*n*)	15.1 (117)	11.8 (33)	23.3 (20)
Diagnosis of PsA, % (*n*)	17.1 (133)	20.8 (58)	19.8 (17)
Use of MTX, % (*n*)	68.5 (532)	64.5 (180)	58.1 (50)
Use of CSs, % (*n*)	66.2 (514)	56.6 (158)	60.5 (52)
Mean DMARDs taken, *n* (s.d.)	1.6 (0.8)	1.5 (0.1)	1.4 (0.7)
Previous biologics, % (*n*)	11.1 (86)	11.8 (33)	
Treatment duration, mean (s.d.), years^d^	5.7 (2.9)	1.9 (1.1)	2.5 (1.1)
ETN biosimilar treatment outcomes			
ETN originator/biosimilar treatment failure, % (*n*)	47.1 (366)	12.2 (34)	11.6 (10)
Time to treatment failure, mean (s.d.), years	3.1 (3.8)	1.01 (1.4)	1.4 (1.9)
Treated in automatic switching area, % (*n*)	72.7 (565)	66.3 (185)	87.2 (75)
DAS-28 pre-treatment, mean (s.d.)^b^	5.1 (1.3)	4.5 (1.1)	<5^a^
DAS-28 post-treatment, mean (s.d.)^b^	3.9 (1.5)	3.7 (1.7)	<5^a^
Difference in DAS-28 pre- and post-treatment, mean(s.d.)^b^	1.2 (1.8)	0.8 (1.5)	<5^a^
GP encounters pre-treatment, mean (s.d.)	62.3 (57.5)	87.9 (56)	69.4 (62.2)
GP encounters post-treatment, mean (s.d.)	87.6 (56.1)	61.7 (50)	78.4 (52.1)
Difference in GP encounters pre- and post-treatment, mean (s.d.)	25.2 (84.5)	26.1 (81.5)	8.3 (83.2)

a Data suppressed to protect anonymity.

b For RA patients only.

c From first mention of inflammatory arthritis in primary care records.

d When no end date is present for ETN originator/ETN biosimilar and no additional drugs have been initiated, patients are assumed to have continued to use the treatment.

Missing data: DAS-28 ETN originator patients: 76 scores present pre- and post-treatment, 90.2% missing; DAS-28 ETN biosimilar patients: 31 scores present pre- and post-treatment, 88.9% missing.

ETN: etanercept; GP: general practitioner.

A total of 863 individuals received the ETN originator. Of these, 777 patients received the ETN originator and not the biosimilar (Supplementary Fig. S1, available at *Rheumatology Advances in Practice* online) and were treated predominantly before 2016, before the ETN biosimilar was launched. After 2016, the use of the ETN biosimilar increased and that of the originator declined. Drug discontinuation by Kaplan–Meier analyses showed similar results between the ETN original and biosimilars (Supplementary Fig. S2, available at *Rheumatology Advances in Practice* online).

Patients who received ETN originator only were significantly younger at diagnosis compared with those receiving biosimilars. They also received the highest number of DMARDs (Table 1). Treatment duration was significantly longer for the ETN originator (5.7 years, s.d.: 2.9 years); however, this was attributable to the product being available for longer. Likewise, treatment failure and time to treatment failure were significantly higher in the ETN originator group (Table 1). For co-morbidities of patients receiving ETN originator or ETN biosimilar, please see Supplementary Table S5, available at *Rheumatology Advances in Practice* online.

### Comparing the use of the ETN biosimilar at automatic *vs* selective LHBs

At the selective use LHB, 105 patients were treated with the ETN biosimilar. Of these, 10.5% (11 of 105) had previously been treated with the ETN originator. This compared with 28.9% (75 of 260) in the automatic use area (difference: 18.4, 95% CI: 9.4, 25.7). There was a significantly higher use of MTX in the automatic area (difference: 13.6, 95% CI: 2.6, 24.5) and a higher use of prior biologics (difference: 20, 95% CI: 9.8, 28.6). Treatment failure was significantly higher in the automatic use area (15 *vs* 4.8%; difference: 10.0, 95% CI: 3.2, 15.8). Following treatment with the ETN biosimilar, there was a significant reduction in DASs pre- and post-treatment (difference: 1.2, 95% CI: 1.0, 1.9) and GP encounters pre- and post-treatment (difference: 35.3, 95% CI: 15.3, 55.4) in the selective area compared with the automatic area (Table 2). For co-morbidities of ETN biosimilar-treated patients within the selective or automatic use area, please see Supplementary Table S6, available at *Rheumatology Advances in Practice* online.

**Table 2:** Patient characteristics and treatment outcomes of etanercept biosimilar-treated patients within the selective or automatic use area

Parameter	**Selective SB4 area** **(*n* = 105)**	**Automatic SB4 area** **(*n* = 260)**	Difference (95% CI)
Female, % (*n*)	58.1 (61)	58.9 (153)	0.8 (−10.1, 11.9)
BMI, mean (s.d.), kg/m^2^	25.9 (4.3)	26.7 (5.9)	0.8 (−0.8, 2.4)
Age at diagnosis, mean (s.d.), years	50.5 (14.3)	52.9 (13.2)	2.4 (−0.7, 5.5)
Disease duration from commencement of ETN biosimilar, mean (s.d.), years^a^	28.7 (17.1)	28.2 (16)	0.5 (−4.4, 3.4)
RA diagnosis, % (*n*)	61.9 (65)	69.6 (181)	7.7 (−2.8, 18.6)
PsA diagnosis, % (*n*)	24.8 (26)	18.8 (49)	5.9 (−15.9, 3.0)
AS diagnosis, % (*n*)	18.1 (19)	13.1 (34)	5.0 (−14.2, 2.7)
Use of MTX, % (*n*)	53.3 (56)	66.9 (174)	13.6 (2.6, 24.5)^*^
Use of CS, % (*n*)	55.2 (58)	58.5 (152)	3.2 (−7.8, 14.4)
Previous csDMARDs, mean (s.d.)	1.6 (0.6)	1.5 (0.6)	0.1 (−0.2, 0.1)
ETN biosimilar treatment duration, mean (s.d.), years^b^	1.8 (0.9)	2.2 (1.2)	0.4 (0.1, 0.7)^*^
Previous ETN originator, % (*n*)	10.5 (11)	28.9 (75)	18.4 (9.4, 25.7)^*^
Previous biologics, % (*n*)	18.1 (19)	38.1 (99)	20 (9.8, 28.6)^*^
ETN biosimilar treatment outcomes			
ETN biosimilar treatment failure, % (*n*)	4.8 (5)	15 (39)	10.2 (3.2, 15.8)^*^
ETN biosimilar treatment failure, with previous ETN use, % (*n*)	0	3.8 (10)	–
ETN biosimilar treatment failure, with no previous ETN use, % (*n*)	4.8 (5)	11.2 (29)	6.4 (11.6, 0.4)
Time to treatment failure, mean (s.d.), years	1.2 (1.5)	1.1 (1.4)	
DAS-28 pre-ETN biosimilar, mean (s.d.)^c^	4.7 (1.4)	4.2 (1.3)	0.5 (0.0, 0.1)
DAS-28 post-ETN biosimilar, mean (s.d.)^c^	3.1 (1.9)	3.8 (1.4)	0.7 (−1.7, 0.3)
Difference in DAS-28 score pre- and post-ETN biosimilar, mean (s.d.)^c^	1.6 (1.8)	0.4 (0.6)	1.2 (1.0, 1.9)^*^
Reduction in GP encounters post-ETN biosimilar, mean (s.d.)	44.1 (69.8)	8.8 (85.6)	35.3 (15.3, 55.4)^*^

a From first mention of inflammatory arthritis in primary care records.

b When no end date is present for ETN originator and no additional drugs have been initiated, patients are assumed to have continued to use the treatment.

c For RA patients only. Missing data: DAS-28 selective area: 17 scores present pre- and post-treatment, 83.8% missing; DAS-28 automatic area: 17 scores present pre- and post-treatment, 93.5% missing.

* *P* *<* 0.05.

csDMARD: conventional synthetic DMARD; ETN: etanercept; GP: general practitioner; SB4: etanercept biosimilar.

When exploring biologic-naïve biosimilar-treated patients only in the selective *vs* automatic use area, the same factors remained significant; MTX use (difference: 17.7, 95% CI: 5, 30) was significantly higher in the automatic use area. For outcomes following treatment with ETN biosimilar, treatment failure (difference: 9.8, 95% CI: 1.3, 17) was significantly higher in the automatic use area. The difference in DAS-28 values pre- and post-ETN biosimilar was significantly less in the automatic area (difference: 0.6, 95% CI: 0.3, 0.9), as was the reduction in primary care encounters post-ETN originator (difference: 32.5, 95% CI: 10.5, 54.5). The only difference was that previous csDMARD use was higher in the automatic area in biologic-naïve patients (difference 12.3, 95% CI: 0.5, 24.4; Table 3).

**Table 3:** Characteristics and treatment outcomes of biologic-naïve etanercept biosimilar-treated patients within the selective or automatic use area

Parameter	**Selective SB4 area** **(*n* = 86)**	**Automatic SB4 area** **(*n* = 160)**	Difference (95% CI)
Female, % (*n*)	54.6 (47)	61.3 (98)	6.7 (−6.1, 19.3)
BMI, mean (s.d.), kg/m^2^	26.1 (4.4)	26.9 (5.5)	0.8 (−2.5, 0.9)
Age at diagnosis, mean (s.d.), years	49.9 (15)	52.7 (13.7)	2.8 (−6.5, 0.9)
Disease duration from commencement of ETN biosimilar, mean (s.d.), years^b^	28.9 (17.1)	27.9 (16.9)	1 (−3.5, 5.5)
RA diagnosis, % (*n*)	86 (74)	91.9 (147)	5.8 (−2, 15.3)
PsA diagnosis, % (*n*)	50 (43)	49.4 (79)	0.6 (−13.5, 12.3)
AS diagnosis, % (*n*)	38.4 (33)	35 (46)	3.4 (−16, 8.9)
Use of MTX, % (*n*)	52.3 (45)	70 (112)	17.7 (5, 30)*
Use of CSs, % (*n*)	53.5 (46)	58.1 (93)	4.6 (−8.1, 17.4)
Previous csDMARDs, mean (s.d.)	64 (55)	76.3 (122)	12.3 (0.5, 24.4)*
ETN biosimilar treatment duration, mean (s.d.), years^c^	1.69 (0.83)	2 (1.1)	0.3 (0.04, 0.6)
Co-morbidities			
Hyperlipidaemia, % (*n*)	10.5 (9)	8.8 (14)	1.7 (−10.7, 5.6)
Hypertension, % (*n*)	25.6 (22)	30 (48)	4.4 (−7.7, 15.4)
Kidney disease, % (*n*)	10.5 (9)	6.3 (10)	4.2 (−12.9, 2.7)
Cardiovascular disease, % (*n*)	<5	7.5 (12)	–
Diabetes, % (*n*)	<5	10.6 (17)	–
Orthopaedic surgery, % (*n*)	16.3 (14)	21.9 (35)	5.6 (−5.2, 15.1)
ETN biosimilar treatment outcomes			
ETN biosimilar treatment failure, % (*n*)	5.8 (5)	15.6 (25)	9.8 (1.3, 17)^*^
Time to treatment failure, mean (s.d.), years	1.3 (1)	1.3 (1)	0 (−0.3, 0.3)
DAS-28 pre-ETN biosimilar, mean (s.d.)^d^	4.1 (1.2)	4.9 (0.8)	0.8 (−1.6, 0)
DAS-28 post-ETN biosimilar, mean (s.d.)^d^	3.1 (2)	4.5 (1.1)	1.4 (0.1, 2.7)
Difference in DAS-28 score pre- and post-ETN biosimilar, mean (s.d.)^d^	1 (1.9)	0.4 (0.8)	0.6 (0.3, 0.9)
Reduction in GP encounters post-ETN biosimilar, mean (s.d.)	50 (70.1)	17.5 (82.4)	32.5 (10.5, 54.5)

a Data suppressed to protect anonymity.

b From first mention of inflammatory arthritis in primary care records.

c When no end date is present for ETN biosimilar or no additional drugs have been initiated, patients are assumed to have continued to use the treatment.

^d^Missing data: DAS-28 selective area: 16 scores present pre- and post-treatment, 81.4% missing; DAS-28 automatic area: 13 scores present pre- and post-treatment, 91.9% missing.

* *P* < 0.05.

csDMARD: conventional synthetic DMARD; ETN: etanercept; GP: general practitioner; SB4: etanercept biosimilar.

### ETN biosimilar treatment failure

The proportion of patients who discontinued ETN biosimilar treatment was 12.1% (44 of 365). For the ETN biosimilar-failure patients, a significantly larger proportion [88.6% (39 of 44)] was treated in the automatic area (difference: 19.8, 95% CI: 6.3, 28.1). GP encounters reduced significantly after treatment in biosimilar-persistent patients compared with treatment-failure patients (difference: 34.7, 95% CI: 19, 50.4). There was also a significantly higher proportion of diabetes cases in the ETN biosimilar-failure patients (15.9%, 7 of 44) compared with the persistent patients (7.2%, 23 of 321; difference: 8.7, 95% CI: 0.1, 22.4; Table 4).

**Table 4:** Characteristics of etanercept biosimilar-failure patients compared with etanercept biosimilar-persistent patients

Parameter	ETN biosimilar-failure patients (*n* = 44)	ETN biosimilar- persistent patients (*n* = 321)	Difference (95% CI)
Female, % (*n*)	63.6 (28)	57.9 (186)	5.7 (−10.0, 19.4)
BMI, mean (s.d.), kg/m^2^	29 (7.7)	26.3 (5.4)	2.7 (−0.3, 5.7)
Age at diagnosis, mean (s.d.), years	52.5 (12.2)	52.2 (13.7)	0.3 (−4.0, 4.6)
Disease duration from commencement of ETN biosimilar, mean (s.d.), years^b^	25.8 (16.8)	28.7 (16.2)	2.9 (−8, 2.2)
Diagnosis of RA, % (*n*)	63.6 (28)	67.9 (218)	4.3 (−9.4, 19.8)
Diagnosis of AS, % (*n*)	<5^a^	15.3 (49)	–
Diagnosis of PsA, % (*n*)	29.5 (13)	19.3 (62)	10.2 (−2.1, 25.4)
Use of MTX, % (*n*)	68.2 (30)	62.3 (200)	5.9 (−9.7, 18.9)
Use of CSs, % (*n*)	65.9 (29)	56.4 (181)	9.5 (−6.2, 22.9)
Previous csDMARDs, mean (s.d.)	1.6 (0.6)	1.5 (0.6)	0.1 (−0.1, 0.3)
ETN biosimilar treatment duration, mean (s.d.), years^c^	1.4 (1.0)	2.2 (1.1)	0.8 (0.5, 1.1)^*^
Previous ETN originator, % (*n*)	22.7 (10)	23.7 (76)	1.0 (−12.0, 14.0)
Previous biologics, % (*n*)	31.8 (14)	32.7 (105)	0.9 (−0.6, 2.4)
Treated in automatic switching area, % (*n*)	88.6 (39)	68.9 (221)	19.8 (6.3, 28.1)^*^
Hyperlipidaemia, % (*n*)	11.4 (5)	7.5 (24)	3.9 (−3.4, 16.7)
Hypertension, % (*n*)	29.5 (13)	28.7 (92)	0.8 (−11.6, 16.3)
Kidney disease, % (*n*)	<5^a^	8.7 (28)	–
Cardiovascular disease, % (*n*)	<5^a^	6.9 (22)	–
Diabetes, % (*n*)	15.9 (7)	7.2 (23)	8.7 (0.1, 22.4)^*^
Orthopaedic surgery, % (*n*)	31.8 (14)	20.2 (65)	11.6 (−1.2, 26.9)
ETN biosimilar treatment outcomes			
DAS-28 pre-ETN biosimilar, mean (s.d.)^d^	4.0 (1.2)	4.4 (1.1)	0.4 (−1.4, 0.6)
DAS-28 post-ETN biosimilar, mean (s.d.)^d^	4.3 (1.2)	3.4 (1.7)	0.9 (−0.6, 2.4)
Difference in DAS-28 pre- and post-ETN biosimilar, mean (s.d.)^d^	0.3 (1.1)	1.0 (1.4)	0.7 (−1.9, 0.5)
GP visits pre-ETN biosimilar, mean (s.d.)	70.4 (45)	85.6 (59.4)	15.2 (−33.5, 3.1)
GP visits post-ETN biosimilar, mean (s.d.)	96 (60.4)	61.3 (48.1)	34.7 (19, 50.4)^*^
Difference in GP encounters pre- and post-ETN biosimilar, mean (s.d.)	25.6 (74)	24.3 (82.6)	1.3 (−24.5, 27.1)

a Data suppressed to protect anonymity.

b From first mention of inflammatory arthritis in primary care records.

c When no end date is present for ETN originator and no additional drugs have been initiated, patients are assumed to have continued to use the treatment.

d For RA patients only.

* *P* < 0.05.

csDMARD: conventional synthetic DMARD; ETN: etanercept; GP: general practitioner.

When comparing ETN originator-persistent patients with ETN biosimilar-persistent patients, significantly more previous biologics were used in the biosimilar-persistent patients (difference: 22.3, 95% CI: 16.5, 28.1). The ETN originator-persistent patients had greater co-morbidities (hypertension, kidney disease and orthopaedic surgery) compared with the ETN biosimilar-persistent patients. There were significantly more GP encounters before treatment in the ETN biosimilar-persistent patients compared with the ETN originator-persistent patients (difference: 26.2, 95% CI: 1.4, 42.0). However, there were significantly fewer GP encounters post-ETN biosimilar in the persistent patients compared with ETN originator patients (difference: 20.1, 95% CI: 7.0, 33.2; Supplementary Table S7, available at *Rheumatology Advances in Practice* online).

When exploring ETN originator and biosimilar failure stratified by time, failure was predominantly at ≥36 months for the ETN originator patients, whereas the majority of patients who failed the ETN biosimilar did so within the first 12 months (Table 5), reflecting their use in different years.

**Table 5:** Etanercept originator and etanercept biosimilar patient failures stratified by treatment duration

Parameter	**ETN originator-failure patients** **(*n* = 366)**	**ETN biosimilar-failure patients** **(*n* = 44)**	Difference (95% CI)
Failed treatment at <1 year, % (*n*)	15.8% (58)	43.2% (19)	27.3 (13.2, 42.3)^*^
Failed treatment between 12 and 24 months, % (*n*)	15.6% (57)	27.3% (12)	11.7 (0.4, 26.7)^*^
Failed treatment at ≥36 months, % (*n*)	68.6% (251)	29.5% (13)	39 (23.6, 51.3)^*^

* *P* < 0.05.

ETN: etanercept.

## Discussion

This study demonstrates that the ETN biosimilar is tolerated well in patients with inflammatory arthritis, with the majority of patients from two LHBs in Wales continuing with treatment.

The 12.1% rate of ETN biosimilar discontinuation observed here is in accordance with those reported from other observational cohort studies that have ranged from 4 to 24% [26]. Like previous studies [17, 21], the retention rate of ETN biosimilar treatment was not different in biologic-naïve or biologic-exposed patients. We observed that the majority of ETN originator failures occurred after ≥36 months of treatment. In contrast, more ETN biosimilar failures were observed within the first 12 months of treatment. This is likely to be attributable to the fact that the ETN originator was the first available biologic used to treat inflammatory arthritis, with alternative biologics unavailable or limited. Therefore, patient adherence is likely to be increased in the absence of an alternative treatment, whereas patients who have been switched to ETN biosimilar might be more likely to discontinue owing to adverse effects or perceived loss of effect and the availability of alternative treatments now. The clinical effectiveness of the ETN biosimilar is supported by the reduction in primary care visits and reduced DASs for both automatic and selective substitution. However, the reductions in GP encounters and disease activity were significantly greater in the selective switching LHB. However, it is worth noting that ETN biosimilar use was higher at the automatic switch LHB. Patients at the automatic switch LHB had significantly increased MTX and biologic use pre-ETN biosimilar. This suggests they had more refractory disease despite no significant difference in DAS between the selective and automatic switch areas.

There was also a significantly higher proportion of diabetes cases in the ETN biosimilar-failure patients, which might reflect that these patients are more prone to adverse events, including infections, and therefore earlier treatment discontinuation. Using the ETN biosimilar in place of the originator product offers cost savings; for example, switching 151 patients in a UK-based hospital resulted in savings of approximately £500 000 per annum [32]. However, despite numerous clinical trials and observational studies demonstrating the equivalent efficacy and safety of ETN biosimilars to ETN originator, concerns regarding the true cost of switching in the event of failure have remained. In fact, previous research has found that patients and rheumatologists alike had concerns about the differences regarding biosimilar efficacy, side effects and suitability. Interestingly, rheumatologists were more likely to have concerns regarding differences between originators and biosimilars, whereas patients trust the decision of the rheumatologist to start or switch to a biosimilar [23]. There is also a lack of clear guidelines regarding the substitution process of ETN biosimilar in the UK. Instead, switching or selective use of the biosimilar might occur on a case-by-case basis and involve clinician and patient preference in the non-mandatory setting. Alternatively, automatic or mandatory switching can be used in other environments (e.g. if made compulsory by the LHB). Our data support the presence of a significant barrier to switching, despite a policy of automatic switching in one LHB; the percentage of patients who were switched from the ETN original to the ETN biosimilar was low (10.5% in the selective switch area and 28.9% in the automatic switch area). This might reflect reluctance of the patients and/or health-care professionals to switch, but further study is required here. For example, investigating the effect of patient counselling and education on biosimilar treatment and its effect on biosimilar adherence will be useful. Previous studies have found ETN biosimilar persistence to be high whether treatment is mandated or not [26, 33], which is a finding observed here. We do, however, find that a small but significantly higher proportion of the ETN biosimilar treatment failures were in patients treated at the automatic switching LHB.

The level of support and patient information regarding ETN biosimilar use is also likely to vary. The factors influencing the acceptance of switching from ETN originator to biosimilar have been reported to be largely sociological in nature, with effects of clinicians and pharmacists being observed [28]. By an increase in patient education and involvement in their treatment decision-making, it is possible that subjective and potentially negative feelings about changes in medication in general [28] could reduce the nocebo effect observed in other studies [14, 15, 26–28] and help to reduce ETN biosimilar withdrawal further.

### Strengths

To our knowledge, our study has the longest follow-up duration of ETN biosimilar patients to date, with >4 years of linked data from 365 patients in a multicentre study (two LHBs, comprising six rheumatology clinics).

This research investigates the effects of differing clinical practice in the same country and reflects the differences in rheumatology care in a real-world setting.

### Limitations

We were unable to access reasons for discontinuation of the ETN originator or biosimilar in individual patients; however, this has been reported elsewhere, with reasons for withdrawal including loss of effect and adverse events [26], disease flares [28, 35] and subjective reasons [15, 26], in addition to a nocebo effect.

There was also no standardized approach to joint decision-making; therefore, we do not know how selective switching was undertaken. Nor do we know what information was provided to patients in order to make an informed decision. Qualitative interviews with health-care professionals would have been helpful to identify barriers to switching.

In our study, more patients were treated overall in the automatic substitution area. Therefore, care must be taken when interpreting these results. The study was also observational in nature, and we can suggest only associations rather than causation.

The use of electronic health data for secondary research purposes carries the risk of incomplete data; therefore, missing data might be an issue. For instance, with the absence of end dates for prescribed medication, the assumption was made that a lack of an alternative biologic meant that ETN originator or ETN biosimilar was continued, with patients persisting on the treatment.

Also, DASs were available for RA, but specific disease activity measures were not available in the records for AS and PsA.

This study relies on routinely collected health data, and as such, we were unable to confirm the findings through qualitative research with the clinicians under the remit of this anonymized study. Despite findings from real-world studies and the gold standard, clinical trial data supporting the safety and efficacy of etanercept biosimilar use [36], some uncertainty remains regarding switching to a biosimilar. This highlights the need for clinical trials in this area that capture reasons for non-adherence to biosimilars comprehensively, in addition to investigating the information or patient counselling provided to patients regarding switching. In this way, the use and adherence to etanercept biosimilars in inflammatory arthritis patients can be analysed fully, to take important contextual factors into account.

Our study demonstrates the effective use of an ETN biosimilar to treat inflammatory arthritis patients in a real-world setting. The biosimilar was well tolerated by patients, with high levels of continuation of ETN biosimilar and positive effects on disease activity and primary care utilization being observed, an effect that was even greater when patients were treated selectively with the drug.

Selective or non-mandatory use of ETN biosimilar appears to be superior in the treatment of inflammatory arthritis patients using the biosimilar. Interventions aimed at increasing patient knowledge about biosimilars might help to mitigate negative experiences of the prospect of switching to a biosimilar and might help to reduce nocebo effects associated with discontinuation observed elsewhere. Further research is required when additional data are available on ETN biosimilar patients and will include a more detailed exploration of ETN biosimilar failure *vs* persistent patients for a greater follow-up duration.

## Supplementary Material

rkac056_Supplementary_Data

## Data Availability

The data underlying this article were provided by SAIL Databank with permission. Data will be shared on request to the corresponding author with permission of SAIL Databank.
