# Supplementary material for: Real-world use of an etanercept biosimilar including selective versus automatic substitution in inflammatory arthritis patients: a UK-based electronic health records study
Source: Rheumatol Adv Pract. 2022 Jul 27;6(2):rkac056. doi: 10.1093/rap/rkac056 (PMC9336562; doi:10.1093/rap/rkac056)
Supplement: rkac056_Supplementary_Data [file rkac056_supplementary_data.zip › Supplementary_Table_S1.docx]

**Supplementary Table S1: SAIL Databank sources for cohort data**

|  | Rheumatology  clinic data | General  (Primary care  Data) | Secondary care  data | Office National Statistics  Death Dataset |
| --- | --- | --- | --- | --- |
| Gender |  |  |  |  |
| Age |  |  |  |  |
| BMI |  |  |  |  |
| Social deprivation quintile |  |  |  |  |
| Smoker |  |  |  |  |
| Alcohol drinker |  |  |  |  |
| RA codes |  |  |  |  |
| AS codes |  |  |  |  |
| PsA codes |  |  |  |  |
| DMARDs |  |  |  |  |
| Biologic agents |  |  |  |  |
| Steroids |  |  |  |  |
| Rheumatology clinic location |  |  |  |  |
| GP visit counts |  |  |  |  |
| Hyperlipidemia |  |  |  |  |
| Hypertension |  |  |  |  |
| Diabetes |  |  |  |  |
| Cardiovascular disease |  |  |  |  |
| Hospitalised with serious infections |  |  |  |  |
| Orthopaedic surgery |  |  |  |  |
| Kidney disease |  |  |  |  |
| Disability payments |  |  |  |  |
| Sick notes issued |  |  |  |  |
| Date of death |  |  |  |  |
