# Supplementary material for: Real-world use of an etanercept biosimilar including selective versus automatic substitution in inflammatory arthritis patients: a UK-based electronic health records study
Source: Rheumatol Adv Pract. 2022 Jul 27;6(2):rkac056. doi: 10.1093/rap/rkac056 (PMC9336562; doi:10.1093/rap/rkac056)
Supplement: rkac056_Supplementary_Data [file rkac056_supplementary_data.zip › Supplementary_Table_S2.docx]

**Supplementary Table S2: Definition of conditions, drugs and events from primary care records using the READ code system**

| READ_CD | DESCRIPTION (CARDIOVASCULAR DISEASE) | | | | | |  |  |  |
| --- | --- | --- | --- | --- | --- | --- | --- | --- | --- |
| 14A3. | H/O: myocardial infarct <60 | | | | | |  |  |  |
| 14A4. | H/O: myocardial infarct >60 | | | | | |  |  |  |
| 14A5. | H/O: angina pectoris | | | | | |  |  |  |
| 14A6. | H/O: heart failure | | | | | |  |  |  |
| 14AH. | H/O: Myocardial infarction in last year | | | | | |  |  |  |
| 14AJ. | H/O: Angina in last year | | | | | |  |  |  |
| 14AM. | H/O: Heart failure in last year | | | | | |  |  |  |
| 14AT. | History of myocardial infarction | | | | | |  |  |  |
| 14AW. | H/O acute coronary syndrome | | | | | |  |  |  |
| 14NB. | H/O: Peripheral vascular disease procedure | | | | | |  |  |  |
| 1J60. | Suspected heart failure | | | | | |  |  |  |
| 1O1.. | Heart failure confirmed | | | | | |  |  |  |
| 21264 | Heart failure resolved | | | | | |  |  |  |
| 323.. | ECG: myocardial infarction | | | | | |  |  |  |
| 3232 | ECG: old myocardial infarction | | | | | |  |  |  |
| 323Z. | ECG: myocardial infarct NOS | | | | | |  |  |  |
| 388D. | New York Heart Assoc classification heart failure symptoms | | | | | |  |  |  |
| 661M5 | Heart failure self-management plan agreed | | | | | |  |  |  |
| 662p. | Heart failure 6 month review | | | | | |  |  |  |
| 662T. | Congestive heart failure monitoring | | | | | |  |  |  |
| 662W. | Heart failure annual review | | | | | |  |  |  |
| 679W1 | Education about deteriorating heart failure | | | | | |  |  |  |
| 679X. | Heart failure education | | | | | |  |  |  |
| 67D4. | Heart failure information given to patient | | | | | |  |  |  |
| 792.. | Coronary artery operations | | | | | |  |  |  |
| 7920 | Saphenous vein graft replacement of coronary artery | | | | | |  |  |  |
| 7921 | Other autograft replacement of coronary artery | | | | | |  |  |  |
| 7922 | Allograft replacement of coronary artery | | | | | |  |  |  |
| 7923 | Prosthetic replacement of coronary artery | | | | | |  |  |  |
| 7924 | Revision of bypass for coronary artery | | | | | |  |  |  |
| 79240 | Revision of bypass for one coronary artery | | | | | |  |  |  |
| 79241 | Revision of bypass for two coronary arteries | | | | | |  |  |  |
| 79242 | Revision of bypass for three coronary arteries | | | | | |  |  |  |
| 79243 | Revision of bypass for four or more coronary arteries | | | | | |  |  |  |
| 7924y | Other specified revision of bypass for coronary artery | | | | | |  |  |  |
| 7924z | Revision of bypass for coronary artery NOS | | | | | |  |  |  |
| 7925 | Connection of mammary artery to coronary artery | | | | | |  |  |  |
| 79275 | Open angioplasty of coronary artery | | | | | |  |  |  |
| 7928 | Transluminal balloon angioplasty of coronary artery | | | | | |  |  |  |
| 79280 | Percut transluminal balloon angioplasty one coronary artery | | | | | |  |  |  |
| 79281 | Percut translum balloon angioplasty mult coronary arteries | | | | | |  |  |  |
| 79282 | Percut translum balloon angioplasty bypass graft coronary a | | | | | |  |  |  |
| 79283 | Percut translum cutting balloon angioplasty coronary artery | | | | | |  |  |  |
| 7928y | Transluminal balloon angioplasty of coronary artery OS | | | | | |  |  |  |
| 7928z | Transluminal balloon angioplasty of coronary artery NOS | | | | | |  |  |  |
| 79290 | Percutaneous transluminal laser coronary angioplasty | | | | | |  |  |  |
| 79293 | Rotary blade coronary angioplasty | | | | | |  |  |  |
| 79294 | Insertion of coronary artery stent | | | | | |  |  |  |
| 79295 | Insertion of drug-eluting coronary artery stent | | | | | |  |  |  |
| 792D. | Other bypass of coronary artery | | | | | |  |  |  |
| 792Dy | Other specified other bypass of coronary artery | | | | | |  |  |  |
| 792Dz | Other bypass of coronary artery NOS | | | | | |  |  |  |
| 793G. | Perc translumin balloon angioplasty stenting coronary artery | | | | | |  |  |  |
| 793Gy | OS perc translumina balloon angioplast stenting coronary art | | | | | |  |  |  |
| 793Gz | Perc translum balloon angioplasty stenting coronary art NOS | | | | | |  |  |  |
| 889A. | Diab mellit insulin-glucose infus acute myocardial infarct | | | | | |  |  |  |
| 8CeC. | Preferred place of care for next exacerbation heart failure | | | | | |  |  |  |
| 8CL3. | Heart failure care plan discussed with patient | | | | | |  |  |  |
| 8CMK. | Has heart failure management plan | | | | | |  |  |  |
| 8CMW8 | Heart failure clinical pathway | | | | | |  |  |  |
| 8H2S. | Admit heart failure emergency | | | | | |  |  |  |
| 8HBE. | Heart failure follow-up | | | | | |  |  |  |
| 8HBJ. | Stroke / transient ischaemic attack referral | | | | | |  |  |  |
| 8L40. | Coronary artery bypass graft operation planned | | | | | |  |  |  |
| 8L41. | Coronary angioplasty planned | | | | | |  |  |  |
| G1yz1 | Rheumatic left ventricular failure | | | | | |  |  |  |
| G232. | Hypertensive heart&renal dis wth (congestive) heart failure | | | | | |  |  |  |
| G3... | Ischaemic heart disease | | | | | |  |  |  |
| G30.. | Acute myocardial infarction | | | | | |  |  |  |
| G301. | Other specified anterior myocardial infarction | | | | | |  |  |  |
| G301z | Anterior myocardial infarction NOS | | | | | |  |  |  |
| G304. | Posterior myocardial infarction NOS | | | | | |  |  |  |
| G305. | Lateral myocardial infarction NOS | | | | | |  |  |  |
| G306. | True posterior myocardial infarction | | | | | |  |  |  |
| G3071 | Acute non-ST segment elevation myocardial infarction | | | | | |  |  |  |
| G308. | Inferior myocardial infarction NOS | | | | | |  |  |  |
| G30B. | Acute posterolateral myocardial infarction | | | | | |  |  |  |
| G30X. | Acute transmural myocardial infarction of unspecif site | | | | | |  |  |  |
| G30X0 | Acute ST segment elevation myocardial infarction | | | | | |  |  |  |
| G30y. | Other acute myocardial infarction | | | | | |  |  |  |
| G30yz | Other acute myocardial infarction NOS | | | | | |  |  |  |
| G30z. | Acute myocardial infarction NOS | | | | | |  |  |  |
| G31.. | Other acute and subacute ischaemic heart disease | | | | | |  |  |  |
| G310. | Postmyocardial infarction syndrome | | | | | |  |  |  |
| G311. | Preinfarction syndrome | | | | | |  |  |  |
| G3110 | Myocardial infarction aborted | | | | | |  |  |  |
| G3111 | Unstable angina | | | | | |  |  |  |
| G3113 | Refractory angina | | | | | |  |  |  |
| G3115 | Acute coronary syndrome | | | | | |  |  |  |
| G31y. | Other acute and subacute ischaemic heart disease | | | | | |  |  |  |
| G31y0 | Acute coronary insufficiency | | | | | |  |  |  |
| G31yz | Other acute and subacute ischaemic heart disease NOS | | | | | |  |  |  |
| G32.. | Old myocardial infarction | | | | | |  |  |  |
| G33.. | Angina pectoris | | | | | |  |  |  |
| G331. | Prinzmetal's angina | | | | | |  |  |  |
| G33z. | Angina pectoris NOS | | | | | |  |  |  |
| G33z3 | Angina on effort | | | | | |  |  |  |
| G33z5 | Post infarct angina | | | | | |  |  |  |
| G33z7 | Stable angina | | | | | |  |  |  |
| G33zz | Angina pectoris NOS | | | | | |  |  |  |
| G34.. | Other chronic ischaemic heart disease | | | | | |  |  |  |
| G340. | Coronary atherosclerosis | | | | | |  |  |  |
| G34y. | Other specified chronic ischaemic heart disease | | | | | |  |  |  |
| G34yz | Other specified chronic ischaemic heart disease NOS | | | | | |  |  |  |
| G34z. | Other chronic ischaemic heart disease NOS | | | | | |  |  |  |
| G35.. | Subsequent myocardial infarction | | | | | |  |  |  |
| G350. | Subsequent myocardial infarction of anterior wall | | | | | |  |  |  |
| G351. | Subsequent myocardial infarction of inferior wall | | | | | |  |  |  |
| G353. | Subsequent myocardial infarction of other sites | | | | | |  |  |  |
| G35X. | Subsequent myocardial infarction of unspecified site | | | | | |  |  |  |
| G36.. | Certain current complication follow acute myocardial infarct | | | | | |  |  |  |
| G38.. | Postoperative myocardial infarction | | | | | |  |  |  |
| G380. | Postoperative transmural myocardial infarction anterior wall | | | | | |  |  |  |
| G381. | Postoperative transmural myocardial infarction inferior wall | | | | | |  |  |  |
| G383. | Postoperative transmural myocardial infarction unspec site | | | | | |  |  |  |
| G384. | Postoperative subendocardial myocardial infarction | | | | | |  |  |  |
| G38z. | Postoperative myocardial infarction, unspecified | | | | | |  |  |  |
| G3y.. | Other specified ischaemic heart disease | | | | | |  |  |  |
| G3z.. | Ischaemic heart disease NOS | | | | | |  |  |  |
| G58.. | Heart failure | | | | | |  |  |  |
| G580. | Congestive heart failure | | | | | |  |  |  |
| G5800 | Acute congestive heart failure | | | | | |  |  |  |
| G5801 | Chronic congestive heart failure | | | | | |  |  |  |
| G5804 | Congestive heart failure due to valvular disease | | | | | |  |  |  |
| G581. | Left ventricular failure | | | | | |  |  |  |
| G5810 | Acute left ventricular failure | | | | | |  |  |  |
| G582. | Acute heart failure | | | | | |  |  |  |
| G583. | Heart failure with normal ejection fraction | | | | | |  |  |  |
| G584. | Right ventricular failure | | | | | |  |  |  |
| G58z. | Heart failure NOS | | | | | |  |  |  |
| G5y4z | Post cardiac operation heart failure NOS | | | | | |  |  |  |
| G670. | Cerebral atherosclerosis | | | | | |  |  |  |
| G677. | Occlusion/stenosis cerebral arts not result cerebral infarct | | | | | |  |  |  |
| G70.. | Atherosclerosis | | | | | |  |  |  |
| G700. | Aortic atherosclerosis | | | | | |  |  |  |
| G701. | Renal artery atherosclerosis | | | | | |  |  |  |
| G70y0 | Carotid artery atherosclerosis | | | | | |  |  |  |
| Gyu3. | [X]Ischaemic heart diseases | | | | | |  |  |  |
| Gyu30 | [X]Other forms of angina pectoris | | | | | |  |  |  |
| Gyu32 | [X]Other forms of acute ischaemic heart disease | | | | | |  |  |  |
| Gyu33 | [X]Other forms of chronic ischaemic heart disease | | | | | |  |  |  |
| Gyu34 | [X]Acute transmural myocardial infarction of unspecif site | | | | | |  |  |  |
| Gyu36 | [X]Subsequent myocardial infarction of unspecified site | | | | | |  |  |  |
| ZV457 | [V]Presence of aortocoronary bypass graft | | | | | |  |  |  |
| ZV458 | [V]Presence of coronary angioplasty implant and graft | | | | | |  |  |  |
| ZV45K | [V]Presence of coronary artery bypass graft | | | | | |  |  |  |
| READ_CD | DESCRIPTION (DIABETES) | | | | | |  |  |  |
| C1001 | Diabetes mellitus, adult onset, no mention of complication | | | | | |  |  |  |
| C1011 | Diabetes mellitus, adult onset, with ketoacidosis | | | | | |  |  |  |
| C1021 | Diabetes mellitus, adult onset, with hyperosmolar coma | | | | | |  |  |  |
| C1031 | Diabetes mellitus, adult onset, with ketoacidotic coma | | | | | |  |  |  |
| C1041 | Diabetes mellitus, adult onset, with renal manifestation | | | | | |  |  |  |
| C1051 | Diabetes mellitus, adult onset, + ophthalmic manifestation | | | | | |  |  |  |
| C1061 | Diabetes mellitus, adult onset, + neurological manifestation | | | | | |  |  |  |
| C1071 | Diabetes mellitus, adult, + peripheral circulatory disorder | | | | | |  |  |  |
| C1072 | Diabetes mellitus, adult with gangrene | | | | | |  |  |  |
| C1074 | NIDDM with peripheral circulatory disorder | | | | | |  |  |  |
| C109. | Non-insulin dependent diabetes mellitus | | | | | |  |  |  |
| C1090 | Non-insulin-dependent diabetes mellitus with renal comps | | | | | |  |  |  |
| C1091 | Non-insulin-dependent diabetes mellitus with ophthalm comps | | | | | |  |  |  |
| C1092 | Non-insulin-dependent diabetes mellitus with neuro comps | | | | | |  |  |  |
| C1093 | Non-insulin-dependent diabetes mellitus with multiple comps | | | | | |  |  |  |
| C1094 | Non-insulin dependent diabetes mellitus with ulcer | | | | | |  |  |  |
| C1095 | Non-insulin dependent diabetes mellitus with gangrene | | | | | |  |  |  |
| C1096 | Non-insulin-dependent diabetes mellitus with retinopathy | | | | | |  |  |  |
| C1097 | Non-insulin dependent diabetes mellitus - poor control | | | | | |  |  |  |
| C1099 | Non-insulin-dependent diabetes mellitus without complication | | | | | |  |  |  |
| C109A | Non-insulin dependent diabetes mellitus with mononeuropathy | | | | | |  |  |  |
| C109B | Non-insulin dependent diabetes mellitus with polyneuropathy | | | | | |  |  |  |
| C109C | Non-insulin dependent diabetes mellitus with nephropathy | | | | | |  |  |  |
| C109D | Non-insulin dependent diabetes mellitus with hypoglyca coma | | | | | |  |  |  |
| C109E | Non-insulin depend diabetes mellitus with diabetic cataract | | | | | |  |  |  |
| C109F | Non-insulin-dependent d m with peripheral angiopath | | | | | |  |  |  |
| C109G | Non-insulin dependent diabetes mellitus with arthropathy | | | | | |  |  |  |
| C109H | Non-insulin dependent d m with neuropathic arthropathy | | | | | |  |  |  |
| C109J | Insulin treated Type 2 diabetes mellitus | | | | | |  |  |  |
| C109K | Hyperosmolar non-ketotic state in type 2 diabetes mellitus | | | | | |  |  |  |
| C10F. | Type 2 diabetes mellitus | | | | | |  |  |  |
| C10F0 | Type 2 diabetes mellitus with renal complications | | | | | |  |  |  |
| C10F1 | Type 2 diabetes mellitus with ophthalmic complications | | | | | |  |  |  |
| C10F2 | Type 2 diabetes mellitus with neurological complications | | | | | |  |  |  |
| C10F3 | Type 2 diabetes mellitus with multiple complications | | | | | |  |  |  |
| C10F4 | Type 2 diabetes mellitus with ulcer | | | | | |  |  |  |
| C10F5 | Type 2 diabetes mellitus with gangrene | | | | | |  |  |  |
| C10F6 | Type 2 diabetes mellitus with retinopathy | | | | | |  |  |  |
| C10F7 | Type 2 diabetes mellitus - poor control | | | | | |  |  |  |
| C10F9 | Type 2 diabetes mellitus without complication | | | | | |  |  |  |
| C10FA | Type 2 diabetes mellitus with mononeuropathy | | | | | |  |  |  |
| C10FB | Type 2 diabetes mellitus with polyneuropathy | | | | | |  |  |  |
| C10FC | Type 2 diabetes mellitus with nephropathy | | | | | |  |  |  |
| C10FD | Type 2 diabetes mellitus with hypoglycaemic coma | | | | | |  |  |  |
| C10FE | Type 2 diabetes mellitus with diabetic cataract | | | | | |  |  |  |
| C10FF | Type 2 diabetes mellitus with peripheral angiopathy | | | | | |  |  |  |
| C10FG | Type 2 diabetes mellitus with arthropathy | | | | | |  |  |  |
| C10FH | Type 2 diabetes mellitus with neuropathic arthropathy | | | | | |  |  |  |
| C10FJ | Insulin treated Type 2 diabetes mellitus | | | | | |  |  |  |
| C10FK | Hyperosmolar non-ketotic state in type 2 diabetes mellitus | | | | | |  |  |  |
| C10FL | Type 2 diabetes mellitus with persistent proteinuria | | | | | |  |  |  |
| C10FM | Type 2 diabetes mellitus with persistent microalbuminuria | | | | | |  |  |  |
| C10FN | Type 2 diabetes mellitus with ketoacidosis | | | | | |  |  |  |
| C10FQ | Type 2 diabetes mellitus with exudative maculopathy | | | | | |  |  |  |
| C10FR | Type 2 diabetes mellitus with gastroparesis | | | | | |  |  |  |
| C10y1 | Diabetes mellitus, adult, + other speciﬁed manifestation | | | | | |  |  |  |
| C10z1 | Diabetes mellitus, adult onset, + unspeciﬁed complication | | | | | |  |  |  |
| L1806 | Pre-existing diabetes mellitus, non-insulin-dependent | | | | | |  |  |  |
| READ_CD | | DESCRIPTION (DIABETES) | | | |  |  |  |  |
| G200. | | Malignant essential hypertens. | | | |  |  |  |  |
| G220. | | Malignant hypertens.renal dis. | | | |  |  |  |  |
| G2100 | | Malig.hypert.heart dis.-no CCF | | | |  |  |  |  |
| G210z | | Malig.hypertens.heart dis. NOS | | | |  |  |  |  |
| G2111 | | Benign hypert.heart dis-+ CCF | | | |  |  |  |  |
| G2400 | | Second.malig.renovasc.hypert. | | | |  |  |  |  |
| G240z | | Secondary malign.hypertens.NOS | | | |  |  |  |  |
| G201. | | Benign essential hypertension | | | |  |  |  |  |
| G221. | | Benign hypertensive renal dis. | | | |  |  |  |  |
| G21z0 | | Hypertens.heart dis.NOS-no CCF | | | |  |  |  |  |
| G24z0 | | Secondary renovasc.hypert. NOS | | | |  |  |  |  |
| G2... | | Hypertensive disease | | | |  |  |  |  |
| G20.. | | Essential hypertension | | | |  |  |  |  |
| G22.. | | Hypertensive renal disease | | | |  |  |  |  |
| G26.. | | Severe hypertensin (NICE 2011) | | | |  |  |  |  |
| G27.. | | Hypertnsn resistnt to drg ther | | | |  |  |  |  |
| G2y.. | | Hypertensive disease OS | | | |  |  |  |  |
| G20z. | | Essential hypertension NOS | | | |  |  |  |  |
| G22z. | | Hypertensive renal disease NOS | | | |  |  |  |  |
| G203. | | Diastolic hypertension | | | |  |  |  |  |
| G232. | | Hypert ht&ren d+(congs)ht fail | | | |  |  |  |  |
| G234. | | Hyp ht&ren d+both(con)h&r fail | | | |  |  |  |  |
| G244. | | Hypertens 2ndry endocrin disor | | | |  |  |  |  |
| G202. | | Systolic hypertension | | | |  |  |  |  |
| G21z1 | | Hypertens.heart dis.NOS- + CCF | | | |  |  |  |  |
| G222. | | Hypertens renal dis+renal fail | | | |  |  |  |  |
| G233. | | Hypertn hrt&ren dis+renal fail | | | |  |  |  |  |
| G24z1 | | Hypertension secondary to drug | | | |  |  |  |  |
| G21zz | | Hypertensive heart disease NOS | | | |  |  |  |  |
| G24zz | | Secondary hypertension NOS | | | |  |  |  |  |
| G21.. | | Hypertensive heart disease | | | |  |  |  |  |
| G2101 | | Malig.hypert.heart dis.-+ CCF | | | |  |  |  |  |
| G2110 | | Benign hypert.heart dis-no CCF | | | |  |  |  |  |
| G211z | | Benign hypertens.heart dis.NOS | | | |  |  |  |  |
| G23.. | | Hypertensive heart+renal dis. | | | |  |  |  |  |
| G24.. | | Secondary hypertension | | | |  |  |  |  |
| G2410 | | Second.benign renovasc.hypert. | | | |  |  |  |  |
| G241z | | Secondary benign hypertens.NOS | | | |  |  |  |  |
| G25.. | | Stge 1 hypertensin (NICE 2011) | | | |  |  |  |  |
| G28.. | | Stge 2 hypertensin (NICE 2011) | | | |  |  |  |  |
| G2z.. | | Hypertensive disease NOS | | | |  |  |  |  |
| G211. | | Benign hypertensive heart dis. | | | |  |  |  |  |
| G231. | | Benign hypert.heart+renal dis. | | | |  |  |  |  |
| G241. | | Secondary benign hypertension | | | |  |  |  |  |
| G251. | | Stage 1 hyp wi ev end org dmge | | | |  |  |  |  |
| G21z. | | Hypertensive heart disease NOS | | | |  |  |  |  |
| G23z. | | Hypertens.heart+renal dis.NOS | | | |  |  |  |  |
| G24z. | | Secondary hypertension NOS | | | |  |  |  |  |
| G210. | | Malignant hypertens.heart dis. | | | |  |  |  |  |
| G230. | | Malig.hypert.heart+renal dis. | | | |  |  |  |  |
| G240. | | Secondary malignant hypertens. | | | |  |  |  |  |
| G250. | | Stage 1 hyp wo ev end org dmge | | | |  |  |  |  |
| READ_CD | | DESCRIPTION (Hyperlipidaemia) | | | | | |  |  |
| C324. | | Hyperlipidaemia NOS | | | | | |  |  |
| C322. | | Mixed hyperlipidaemia | | | | | |  |  |
| C3201 | | Hyperbetalipoproteinaemia | | | | | |  |  |
| C3203 | | LDL hyperlipoproteinaemia | | | | | |  |  |
| C320. | | Pure hypercholesterolaemia | | | | | |  |  |
| C320y | | Pure hypercholesterolaemia OS | | | | | |  |  |
| C3200 | | Familial hypercholesterolaemia | | | | | |  |  |
| C320z | | Pure hypercholesterolaemia NOS | | | | | |  |  |
| C3202 | | Hyperlipidaemia, group A | | | | | |  |  |
| C3206 | | Polygenic hypercholesterolemia | | | | | |  |  |
| C3205 | | Fam defect apolipoprot B-100 | | | | | |  |  |
| C3204 | | Fredrickson type IIa lipidaem | | | | | |  |  |
| C328. | | Dyslipidaemia | | | | | |  |  |
| C321. | | Pure hyperglyceridaemia | | | | | |  |  |
| C3210 | | Hypertriglyceridaemia | | | | | |  |  |
| READ_CD | | DESCRIPTION (DMARD) | | | | | |  |  |
| ej26. | | Chloroquine phosphate 250mg tablets | | | | | |  |  |
| ej25. | | Nivaquine 272.5mg(200mg base)/5ml Injection (Aventis Pharma) | | | | | |  |  |
| ejC.. | | Chloroquine phosphate 250mg tablets and Proguanil 100mg tablets | | | | | |  |  |
| j54z. | | Hydroxychloroquine 200mg tablets | | | | | |  |  |
| ej24. | | Nivaquine 68mg/5ml Oral solution (Aventis Pharma) | | | | | |  |  |
| ej23. | | Nivaquine 200mg Tablet (Aventis Pharma) | | | | | |  |  |
| ej21. | | Avloclor 250mg tablets (AstraZeneca UK Ltd) | | | | | |  |  |
| ej31. | | Plaquenil 200mg tablets (Sanofi) | | | | | |  |  |
| j541. | | Plaquenil 200mg tablets (Sanofi) | | | | | |  |  |
| ej2w. | | Chloroquine phosphate 80mg/5ml oral solution | | | | | |  |  |
| ej22. | | Malarivon 80mg/5ml syrup (Wallace Manufacturing Chemists Ltd) | | | | | |  |  |
| j542. | | Quinoric 200mg tablets (Bristol Laboratories Ltd) | | | | | |  |  |
| h871. | | Adalimumab 40mg injection | | | | | |  |  |
| h873. | | Adalimumab 40mg injection | | | | | |  |  |
| h89w. | | Enbrel 25mg powder and solvent for solution for injection vials (Pfizer Ltd) | | | | | |  |  |
| h892. | | Etanercept 25mg powder and solvent for solution for injection vials | | | | | |  |  |
| h8Bz. | | Infliximab 100mg powder for solution for infusion vials | | | | | |  |  |
| h89z. | | Enbrel 50mg powder and solvent for solution for injection vials (Wyeth Pharmaceuticals) | | | | | |  |  |
| h8B1. | | Remicade 100mg powder for solution for infusion vials (Merck Sharp & Dohme Ltd) | | | | | |  |  |
| h872. | | Humira 40mg Injection (Abbott Laboratories Ltd) | | | | | |  |  |
| h874. | | Humira 40mg Injection (Abbott Laboratories Ltd) | | | | | |  |  |
| h891. | | Etanercept 50mg powder and solvent for solution for injection vials | | | | | |  |  |
| h893. | | Etanercept 50mg injection solution | | | | | |  |  |
| h895. | | Etanercept 50mg injection solution | | | | | |  |  |
| h89v. | | Enbrel 25mg/0.5ml solution for injection pre-filled syringes (Pfizer Ltd) | | | | | |  |  |
| h894. | | Etanercept 25mg/0.5ml solution for injection pre-filled syringes | | | | | |  |  |
| h89u. | | Enbrel 50mg Solution for injection (Pfizer Consumer Healthcare Ltd) | | | | | |  |  |
| h89x. | | Enbrel 50mg Solution for injection (Pfizer Consumer Healthcare Ltd) | | | | | |  |  |
| h89y. | | Enbrel Paediatric 25mg powder and solvent for solution for injection vials (Pfizer Ltd) | | | | | |  |  |
| h8G2. | | Cimzia 200mg/1ml solution for injection pre-filled syringes (UCB Pharma Ltd) | | | | | |  |  |
| h8G1. | | Certolizumab pegol 200mg/1ml solution for injection pre-filled syringes | | | | | |  |  |
| h71z. | | Azathioprine 50mg powder for solution for injection vials | | | | | |  |  |
| h71y. | | Azathioprine 25mg tablets | | | | | |  |  |
| h71x. | | Azathioprine 50mg tablets | | | | | |  |  |
| h712. | | Imuran 25mg Tablet (Wellcome Medical Division) | | | | | |  |  |
| h713. | | Imuran 50mg Tablet (Wellcome Medical Division) | | | | | |  |  |
| h711. | | Azamune 50mg Tablet (Penn Pharmaceuticals Ltd) | | | | | |  |  |
| h718. | | Azathioprine 10mg tablets | | | | | |  |  |
| h714. | | Imuran 50mg powder for solution for injection vials (Aspen Pharma Trading Ltd) | | | | | |  |  |
| h717. | | Oprisine 50mg Tablet (Opus Pharmaceuticals Ltd) | | | | | |  |  |
| h715. | | Immunoprin 50mg tablets (Ashbourne Pharmaceuticals Ltd) | | | | | |  |  |
| h716. | | Berkaprine 50mg Tablet (Rorer Pharmaceuticals Ltd) | | | | | |  |  |
| h719. | | Imuran 10mg Tablet (Wellcome Medical Division) | | | | | |  |  |
| hh11. | | Rituximab 100mg/10ml solution for infusion vials | | | | | |  |  |
| hh12. | | Rituximab 500mg/50ml solution for infusion vials | | | | | |  |  |
| h14.. | | Cyclophosphamide | | | | | |  |  |
| h146. | | Endoxana 10mg tablet | | | | | |  |  |
| h141. | | Cyclophosphamide 50mg tablet | | | | | |  |  |
| h147. | | Endoxana 50mg tablet | | | | | |  |  |
| h142. | | Cyclophosphamide 100mg injection (pdr for recon) | | | | | |  |  |
| h148. | | Endoxana 100mg injection (pdr for recon) | | | | | |  |  |
| h143. | | Cyclophosphamide 200mg injection (pdr for recon) | | | | | |  |  |
| h14A. | | Cyclophos 200mg injection (pdr for recon) | | | | | |  |  |
| h149. | | Endoxana 200mg injection (pdr for recon) | | | | | |  |  |
| h144. | | Cyclophosphamide 500mg injection (pdr for recon) | | | | | |  |  |
| h14B. | | Cyclophos 500mg injection (pdr for recon) | | | | | |  |  |
| h145. | | Cyclophosphamide 1g injection (pdr for recon) | | | | | |  |  |
| h14C. | | Cyclophos 1g injection (pdr for recon) | | | | | |  |  |
| x00Nb | | Cyclophosphamide 1g/50mL infusion | | | | | |  |  |
| x00Na | | Cyclophosphamide 2g/100mL infusion | | | | | |  |  |
| x00NZ | | Cyclophosphamide 4g/200mL infusion | | | | | |  |  |
| h82A. | | Neoral 25mg capsules (Novartis Pharmaceuticals UK Ltd) | | | | | |  |  |
| h82C. | | Neoral 100mg capsules (Novartis Pharmaceuticals UK Ltd) | | | | | |  |  |
| h82x. | | Ciclosporin 100mg/ml oral solution sugar free | | | | | |  |  |
| h82D. | | Neoral 100mg/ml oral solution (Novartis Pharmaceuticals UK Ltd) | | | | | |  |  |
| h829. | | Ciclosporin 50mg capsules | | | | | |  |  |
| h826. | | Ciclosporin 25mg capsules | | | | | |  |  |
| h827. | | Ciclosporin 100mg capsules | | | | | |  |  |
| h824. | | Sandimmun 25mg capsules (Novartis Pharmaceuticals UK Ltd) | | | | | |  |  |
| h82B. | | Neoral 50mg capsules (Novartis Pharmaceuticals UK Ltd) | | | | | |  |  |
| h821. | | Sandimmun 100mg/ml oral solution (Novartis Pharmaceuticals UK Ltd) | | | | | |  |  |
| h825. | | Sandimmun 100mg capsules (Novartis Pharmaceuticals UK Ltd) | | | | | |  |  |
| h828. | | Sandimmun 50mg capsules (Novartis Pharmaceuticals UK Ltd) | | | | | |  |  |
| h82E. | | Ciclosporin 10mg capsules | | | | | |  |  |
| h82F. | | Neoral 10mg capsules (Novartis Pharmaceuticals UK Ltd) | | | | | |  |  |
| h822. | | Sandimmun 50mg/ml Concentrate for solution for infusion (Novartis Pharmaceuticals UK Ltd) | | | | | |  |  |
| h82y. | | Ciclosporin 50mg/1ml solution for infusion ampoules | | | | | |  |  |
| h82I. | | Deximune 50mg capsules (Dexcel-Pharma Ltd) | | | | | |  |  |
| h82J. | | Deximune 100mg capsules (Dexcel-Pharma Ltd) | | | | | |  |  |
| h82H. | | Deximune 25mg capsules (Dexcel-Pharma Ltd) | | | | | |  |  |
| h82z. | | Ciclosporin 250mg/5ml solution for infusion ampoules | | | | | |  |  |
| h82.. | | Ciclosporin product | | | | | |  |  |
| h82G. | | Sangcya 100mg/mL oral solution | | | | | |  |  |
| h823. | | Sandimmun 250mg/5mL oily infusion concentrate | | | | | |  |  |
| h82K. | | CAPIMUNE 25mg capsules | | | | | |  |  |
| h82L. | | CAPIMUNE 50mg capsules | | | | | |  |  |
| h82M. | | CAPIMUNE 100mg capsules | | | | | |  |  |
| h82N. | | CAPSORIN 25mg capsules | | | | | |  |  |
| h82O. | | CAPSORIN 50mg capsules | | | | | |  |  |
| h82P. | | CAPSORIN 100mg capsules | | | | | |  |  |
| j513. | | Myocrisin 10mg/0.5ml solution for injection ampoules (Sanofi) | | | | | |  |  |
| j515. | | Myocrisin 50mg/0.5ml solution for injection ampoules (Sanofi) | | | | | |  |  |
| j514. | | Myocrisin 20mg/0.5ml solution for injection ampoules (Sanofi) | | | | | |  |  |
| j51z. | | Sodium aurothiomalate 50mg/0.5ml solution for injection ampoules | | | | | |  |  |
| j51x. | | Sodium aurothiomalate 10mg/0.5ml solution for injection ampoules | | | | | |  |  |
| j51y. | | Sodium aurothiomalate 20mg/0.5ml solution for injection ampoules | | | | | |  |  |
| x01J9 | | Hydroxychloroquine | | | | | |  |  |
| ej3.. | | Hydroxychloroquine sulphate [anti malarial] | | | | | |  |  |
| j54.. | | Hydroxychloroquine sulphate [anti- rheumatic] | | | | | |  |  |
| h862. | | Kineret 100mg/0.67ml solution for injection pre-filled syringes (Swedish Orphan Biovitrum Ltd) | | | | | |  |  |
| h861. | | Anakinra 100mg/0.67ml solution for injection pre-filled syringes | | | | | |  |  |
| h8Fz. | | Tocilizumab 80mg/4ml solution for infusion vials | | | | | |  |  |
| h8Fy. | | Tocilizumab 200mg/10ml solution for infusion vials | | | | | |  |  |
| j59z. | | Leflunomide 100mg tablets | | | | | |  |  |
| j59x. | | Leflunomide 10mg tablets | | | | | |  |  |
| j59y. | | Leflunomide 20mg tablets | | | | | |  |  |
| j591. | | Arava 10mg tablets (Sanofi) | | | | | |  |  |
| j592. | | Arava 20mg tablets (Sanofi) | | | | | |  |  |
| j593. | | Arava 100mg tablets (Sanofi) | | | | | |  |  |
| h34.. | | METHOTREXATE | | | | | |  |  |
| h341. | | METHOTREXATE 2.5mg tablets | | | | | |  |  |
| h342. | | METHOTREXATE 10mg tablets | | | | | |  |  |
| h343. | | METHOTREXATE 2.5mg/1mL solution for injection | | | | | |  |  |
| h344. | | METHOTREXATE 5mg/2mL solution for injection | | | | | |  |  |
| h345. | | METHOTREXATE 25mg/1mL solution for injection | | | | | |  |  |
| h346. | | METHOTREXATE 50mg/2mL solution for injection | | | | | |  |  |
| h347. | | METHOTREXATE 100mg/4mL injection solution | | | | | |  |  |
| h348. | | METHOTREXATE 200mg/8mL solution for injection | | | | | |  |  |
| h349. | | METHOTREXATE 500mg/20mL solution for injection | | | | | |  |  |
| h34A. | | METHOTREXATE 15mg/1.5mL solution for injection prefilled syringe | | | | | |  |  |
| h34B. | | METHOTREXATE 20mg/2mL solution for injection prefilled syringe | | | | | |  |  |
| h34C. | | METHOTREXATE 25mg/2.5mL solution for injection prefilled syringe | | | | | |  |  |
| h34D. | | METOJECT 7.5mg/0.75mL solution for injection prefilled syringe | | | | | |  |  |
| h34E. | | METOJECT 10mg/1mL solution for injection prefilled syringe | | | | | |  |  |
| h34F. | | METOJECT 15mg/1.5mL solution for injection prefilled syringe | | | | | |  |  |
| h34G. | | METOJECT 20mg/2mL solution for injection prefilled syringe | | | | | |  |  |
| h34H. | | METOJECT 25mg/2.5mL solution for injection prefilled syringe | | | | | |  |  |
| h34i. | | EMTEXATE 1g/40mL solution for injection | | | | | |  |  |
| h34j. | | EMTEXATE 5g/200mL solution for injection | | | | | |  |  |
| h34k. | | EMTEXATE 1g/10mL solution for injection | | | | | |  |  |
| h34L. | | METOJECT 7.5mg/0.15mL solution for injection pfs | | | | | |  |  |
| h34M. | | METHOTREXATE 7.5mg/0.15mL solution for injection pfs | | | | | |  |  |
| h34N. | | METOJECT 10mg/0.2mL solution for injection prefilled syringe | | | | | |  |  |
| h34O. | | METHOTREXATE 10mg/0.2mL solution for injection pfs | | | | | |  |  |
| h34P. | | METOJECT 15mg/0.3mL solution for injection prefilled syringe | | | | | |  |  |
| h34Q. | | METHOTREXATE 15mg/0.3mL solution for injection pfs | | | | | |  |  |
| h34R. | | METOJECT 20mg/0.4mL solution for injection prefilled syringe | | | | | |  |  |
| h34S. | | METHOTREXATE 20mg/0.4mL solution for injection pfs | | | | | |  |  |
| h34T. | | METOJECT 25mg/0.5mL solution for injection prefilled syringe | | | | | |  |  |
| h34U. | | METHOTREXATE 25mg/0.5mL solution for injection pfs | | | | | |  |  |
| h34V. | | METOJECT 30mg/0.6mL solution for injection prefilled syringe | | | | | |  |  |
| h34W. | | METHOTREXATE 30mg/0.6mL solution for injection pfs | | | | | |  |  |
| h34X. | | EBETREX 7.5mg/0.75mL soln for injection prefilled syringe | | | | | |  |  |
| h34Y. | | EBETREX 10mg/1mL solution for injection prefilled syringe | | | | | |  |  |
| h34Z. | | EBETREX 15mg/1.5mL solution for injection prefilled syringe | | | | | |  |  |
| h3G1. | | EBETREX 20mg/1mL solution for injection prefilled syringe | | | | | |  |  |
| h3G2. | | EBETREX 25mg/1.25mL solution for injection prefilled syringe | | | | | |  |  |
| h3G3. | | EBETREX 30mg/1.5mL solution for injection prefilled syringe | | | | | |  |  |
| h3G4. | | METHOTREXATE 20mg/1mL solution for injection p/f syringe | | | | | |  |  |
| h3G5. | | METHOTREXATE 25mg/1.25mL solution for injection p/f syringe | | | | | |  |  |
| h3G6. | | METHOTREXATE 30mg/1.5mL solution for injection p/f syringe | | | | | |  |  |
| h3G7. | | METOJECT 12.5mg/0.25mL soln for injection prefilled syringe | | | | | |  |  |
| h3G8. | | METHOTREXATE 12.5mg/0.25mL solution for injection pfs | | | | | |  |  |
| h3G9. | | METOJECT 17.5mg/0.35mL soln for injection prefilled syringe | | | | | |  |  |
| h3GA. | | METHOTREXATE 17.5mg/0.35mL solution for injection pfs | | | | | |  |  |
| h3GB. | | METOJECT 22.5mg/0.45mL soln for injection prefilled syringe | | | | | |  |  |
| h3GC. | | METHOTREXATE 22.5mg/0.45mL solution for injection pfs | | | | | |  |  |
| h3GD. | | METOJECT 27.5mg/0.55mL soln for injection prefilled syringe | | | | | |  |  |
| h3GE. | | METHOTREXATE 27.5mg/0.55mL solution for injection pfs | | | | | |  |  |
| j561. | | Auranofin 3mg tablets | | | | | |  |  |
| j562. | | Ridaura Tiltab 3mg tablets (Astellas Pharma Ltd) | | | | | |  |  |
| j521. | | Penicillamine 50mg tablets | | | | | |  |  |
| j523. | | Penicillamine 250mg tablets | | | | | |  |  |
| j522. | | Penicillamine 125mg tablets | | | | | |  |  |
| j525. | | Distamine 125mg tablets (Alliance Pharmaceuticals Ltd) | | | | | |  |  |
| j526. | | Distamine 250mg tablets (Alliance Pharmaceuticals Ltd) | | | | | |  |  |
| j524. | | Distamine 50mg Tablet (Alliance Pharmaceuticals Ltd) | | | | | |  |  |
| j528. | | Pendramine 250mg Tablet (Viatris Pharmaceuticals Ltd) | | | | | |  |  |
| j527. | | Pendramine 125mg Tablet (Viatris Pharmaceuticals Ltd) | | | | | |  |  |
| aa6z. | | Sulfasalazine 3g/100ml enema | | | | | |  |  |
| aa64. | | Salazopyrin 3g/100ml Enema (Pharmacia Ltd) | | | | | |  |  |
| aa62. | | Salazopyrin EN-Tabs 500mg (Pfizer Ltd) | | | | | |  |  |
| j551. | | Salazopyrin EN-Tabs 500mg (Pfizer Ltd) | | | | | |  |  |
| aa6v. | | Sulfasalazine 500mg gastro-resistant tablets | | | | | |  |  |
| aa61. | | Salazopyrin 500mg Tablet (Pharmacia Ltd) | | | | | |  |  |
| aa6y. | | Sulfasalazine 500mg tablet | | | | | |  |  |
| j55z. | | Sulfasalazine 500mg suppositories | | | | | |  |  |
| aa63. | | Salazopyrin 500mg Suppository (Pharmacia Ltd) | | | | | |  |  |
| aa6u. | | Sulfasalazine 250mg/5ml oral solution | | | | | |  |  |
| aa65. | | Salazopyrin 250mg/5ml oral suspension (Pfizer Ltd) | | | | | |  |  |
| aa66. | | Sulfasalazine 500mg gastro-resistant tablets (Actavis UK Ltd) | | | | | |  |  |
| j552. | | Sulazine EC 500mg tablets (Genesis Pharmaceuticals Ltd) | | | | | |  |  |
| READ_CD | | DESCRIPTION (SICKNOTE) | | | | | |  |  |
| 9DK.. | | Sick note generated from secondary care done by GP practice | | | | | |  |  |
| 9C8.. | | Sickness notification of GP | | | | | |  |  |
| 9C83. | | Sickness payment record | | | | | |  |  |
| 13O.. | | Sickness benefit | | | | | |  |  |
| 13O1. | | Sickness benefit | | | | | |  |  |
| 13O2. | | Statutory sick pay | | | | | |  |  |
| 9K8.. | | Sickness certificate | | | | | |  |  |
| READ_CD | | | | DESCRIPTION (STEROIDS) |  |  | | | |
| fe43. | | | | *HYDROCORTISTAB 20mg tablets | | | | | |
| fe93. | | | | *DEFLAZACORT 30mg tablets | | | | | |
| fe3A. | | | | DEXSOL 2mg/5mL oral solution | | | | | |
| fe31. | | | | DEXAMETHASONE 500micrograms tablets | | | | | |
| fe6k. | | | | PREDNISOLONE 50mg tablets | | | | | |
| fe37. | | | | *ORADEXON 2mg tablets | | | | | |
| fe3r. | | | | DEXAMETHASONE 500micrograms/5mL solution | | | | | |
| fe66. | | | | DELTACORTRIL ENTERIC 5mg tablets | | | | | |
| fe6a. | | | | *DELTASTAB 5mg tablets | | | | | |
| fe6h. | | | | PREDNISOLONE 2.5mg e/c tablets | | | | | |
| fe64. | | | | *DELTA-PHORICOL 5mg tablets | | | | | |
| fe24. | | | | *CORTISTAB 5mg tablets | | | | | |
| fe95. | | | | *DEFLAZACORT 1mg tablets | | | | | |
| fe5p. | | | | METHYLPREDNISOLONE 16mg tablets | | | | | |
| fe6e. | | | | PRECORTISYL FORTE 25mg tablets | | | | | |
| fe94. | | | | *CALCORT 30mg tablets | | | | | |
| fe21. | | | | *CORTISONE 5mg tablets | | | | | |
| fe1y. | | | | BETAMETHASONE 500microgram tablets | | | | | |
| fe1x. | | | | BETAMETHASONE 500micrograms soluble tablets | | | | | |
| fe32. | | | | DEXAMETHASONE 2mg tablets | | | | | |
| fe3u. | | | | DEXAMETHASONE 2mg/5mL liquid | | | | | |
| fe5o. | | | | METHYLPREDNISOLONE 4mg tablets | | | | | |
| fe65. | | | | DELTACORTRIL ENTERIC 2.5mg tablets | | | | | |
| fe6d. | | | | *PRECORTISYL 5mg tablets | | | | | |
| fe22. | | | | CORTISONE 25mg tablets | | | | | |
| fb11. | | | | FLORINEF 100micrograms tablets | | | | | |
| fe25. | | | | *CORTISTAB 25mg tablets | | | | | |
| fe6j. | | | | PREDNISOLONE 5mg soluble tablets | | | | | |
| fe68. | | | | *DELTALONE 5mg tablets | | | | | |
| fe96. | | | | *CALCORT 1mg tablets | | | | | |
| fe41. | | | | HYDROCORTISONE 10mg tablets | | | | | |
| fe69. | | | | *DELTASTAB 1mg tablets | | | | | |
| fe53. | | | | MEDRONE 16mg tablets | | | | | |
| fe5m. | | | | METHYLPREDNISOLONE 100mg tablets | | | | | |
| fe6i. | | | | PREDNISOLONE 5mg e/c tablets | | | | | |
| fe42. | | | | HYDROCORTISONE 20mg tablets | | | | | |
| fe11. | | | | BETNELAN 500micrograms tablets | | | | | |
| fe5n. | | | | METHYLPREDNISOLONE 2mg tablets | | | | | |
| fe23. | | | | *CORTELAN 25mg tablets | | | | | |
| fe12. | | | | BETNESOL 500micrograms tablets | | | | | |
| fe6z. | | | | PREDNISOLONE 25mg tablets | | | | | |
| fe9.. | | | | DEFLAZACORT | | | | | |
| fe2.. | | | | CORTISONE ACETATE | | | | | |
| fe45. | | | | *HYDROCORTONE 20mg tablets | | | | | |
| fe6v. | | | | *PREDNISOLONE 2.5mg tablets | | | | | |
| fe3s. | | | | DEXAMETHASONE 2mg/5mL sugar free solution | | | | | |
| fe67. | | | | *DELTALONE 1mg tablets | | | | | |
| fe92. | | | | CALCORT 6mg tablets | | | | | |
| fe36. | | | | *ORADEXON 500microgram tablets | | | | | |
| fe44. | | | | *HYDROCORTONE 10mg tablets | | | | | |
| fe62. | | | | PREDNISOLONE 5mg tablets | | | | | |
| fe5f. | | | | MEDRONE 100mg tablets | | | | | |
| fe33. | | | | DECADRON 500micrograms tablets | | | | | |
| fe26. | | | | *CORTISYL 25mg tablets | | | | | |
| fe6c. | | | | *PRECORTISYL 1mg tablets | | | | | |
| fb1.. | | | | FLUDROCORTISONE ACETATE | | | | | |
| fb1z. | | | | FLUDROCORTISONE ACET 100microgram tablets | | | | | |
| fe6f. | | | | *PREDNESOL 5mg tablets | | | | | |
| fe6g. | | | | *SINTISONE 5mg tablets | | | | | |
| fe52. | | | | MEDRONE 4mg tablets | | | | | |
| fe91. | | | | DEFLAZACORT 6mg tablets | | | | | |
| fe51. | | | | MEDRONE 2mg tablets | | | | | |
| fe61. | | | | PREDNISOLONE 1mg tablets | | | | | |
