# Supplementary material for: Real-world use of an etanercept biosimilar including selective versus automatic substitution in inflammatory arthritis patients: a UK-based electronic health records study
Source: Rheumatol Adv Pract. 2022 Jul 27;6(2):rkac056. doi: 10.1093/rap/rkac056 (PMC9336562; doi:10.1093/rap/rkac056)
Supplement: rkac056_Supplementary_Data [file rkac056_supplementary_data.zip › Supplementary_Table_S3.docx]

**Supplementary Table S3: Definition of serious infections from secondary care records using the International Classification of Diseases- 10^th^ Revision**

| **ICD-10 Codes** | | | **Upper respiratory tract infections** | | |
| --- | --- | --- | --- | --- | --- |
| J02.0 | | | Streptoccocal sore throat | | |
| J00 | | | Acute nasopharyngitis (common cold) | | |
| J01.90 | | | Acute sinusitis, unspecified | | |
| J02.9 | | | Acute phartngitis | | |
| J03.90 | | | Acute tonsilitis | | |
| J06.9 | | | Acute upper respiratory infections of unspecified site | | |
| J31.0 | | | Chronic rhinitis | | |
| J32.9 | | | Unspecified sinusitis (chronic) | | |
| R05 | | | Cough | | |
|  | | | **Lower respiratory tract infections** | | |
| A37.90 | | | Whooping cough, unpecified organism | | |
| B97.4 | | | Respiratory syncytial virus | | |
| J41.0 | | | Simple chronic bronchitis | | |
| J41.1 | | | Micropurulent chronic bronchitis | | |
| J41.8 | | | Mixed simple and micropurulent chronic bronchitis | | |
| J42.0 | | | Unspecified chronic bronchitis | | |
| J14 | | | Pneumonia due to Haemophilus influenzae | | |
| J15.0 | | | Pneumonia due to Klebsiella pneumoniae | | |
| J15.1 | | | Pneumonia due to Pseudomonas | | |
| J15.2 | | | Pneumonia due to staphylococcus | | |
| J15.3 | | | Pneumonia due to streptococcus, group B | | |
| J15.4 | | | Pneumonia due to other streptococci | | |
| J15.5 | | | Pneumonia due to Escherichia coli | | |
| J15.6 | | | Pneumonia due to other aerobic Gram-negative bacteria | | |
| J15.7 | | | Pneumonia due to Mycoplasma pneumoniae | | |
| J15.8 | | | Other bacterial pneumonia | | |
| J15.9 | | | Bacterial pneumonia, unspecified | | |
| J18.0 | | | Bronchopneumonia, unspecified | | |
| J18.1 | | | Lobar pneumonia, unspecified | | |
| J18.2 | | | Hypostatic pneumonia, unspecified | | |
| J18.8 | | | Other pneumonia, organism unspecified | | |
| J18.9 | | | Pneumonia, unspecified | | |
| R09.1 | | | Pleurisy | | |
|  | | | **Tuberculosis** | | |
| A15.0 | | | Tuberculosis of lung, confirmed by sputum microscopy with or without culture | | |
| A15.1 | | | Tuberculosis of lung, confirmed by culture only | | |
| A15.2 | | | Tuberculosis of lung, confirmed histologically | | |
| A15.3 | | | Tuberculosis of lung, confirmed by unspecified means | | |
| A15.4 | | | Tuberculosis of intrathoracic lymph nodes, confirmed bacteriologically and histologically | | |
| A15.5 | | | Tuberculosis of larynx, trachea and bronchus, confirmed bacteriologically and histologically | | |
| A15.6 | | | Tuberculous pleurisy, confirmed bacteriologically and histologically | | |
| A15.7 | | | Primary respiratory tuberculosis, confirmed bacteriologically and histologically | | |
| A15.8 | | | Other respiratory tuberculosis, confirmed bacteriologically and histologically | | |
| A15.9 | | | Respiratory tuberculosis unspecified, confirmed bacteriologically and histologically | | |
| A16.2 | | | Tuberculosis of lung, without mention of bacteriological or histological confirmation | | |
| A16.3 | | | Tuberculosis of intrathoracic lymph nodes, without mention of bacteriological or histological confirmation | | |
| A16.4 | | | Tuberculosis of larynx, trachea and bronchus, without mention of bacteriological or histological confirmation | | |
| A16.5 | | | Tuberculous pleurisy, without mention of bacteriological or histological confirmation | | |
| A16.7 | | | Primary respiratory tuberculosis without mention of bacteriological or histological confirmation | | |
| A16.8 | | | Other respiratory tuberculosis, without mention of bacteriological or histological confirmation | | |
| A16.9 | | | Respiratory tuberculosis unspecified, without mention of bacteriological or histological confirmation | | |
| A17.0 | | | Tuberculous meningitis | | |
| A17.1 | | | Meningeal tuberculoma | | |
| A17.8 | | | Other tuberculosis of nervous system | | |
| A17.9 | | | Tuberculosis of nervous system, unspecified | | |
| A18.0 | | | Tuberculosis of bones and joints | | |
| A18.1 | | | Tuberculosis of genitourinary system | | |
| A18.2 | | | Tuberculous peripheral lymphadenopathy | | |
| A18.3 | | | Tuberculosis of intestines, peritoneum and mesenteric glands | | |
| A18.4 | | | Tuberculosis of skin and subcutaneous tissue | | |
| A18.5 | | | Tuberculosis of eye | | |
| A18.6 | | | Tuberculosis of ear | | |
| A18.7 | | | Tuberculosis of adrenal glands | | |
| A18.8 | | | Tuberculosis of other specified organs | | |
| A19.0 | | | Acute miliary tuberculosis of a single specified site | | |
| A19.1 | | | Acute miliary tuberculosis of multiple sites | | |
| A19.2 | | | Acute miliary tuberculosis, unspecified | | |
| A19.8 | | | Other miliary tuberculosis | | |
| A19.9 | | | Miliary tuberculosis, unspecified | | |
|  | | | **GI infections** | | |
| A03.0 | | | Shigellosis due to Shigella dysenteriae | | |
| A03.1 | | | Shigellosis due to Shigella flexneri | | |
| A03.2 | | | Shigellosis due to Shigella boydii | | |
| A03.3 | | | Shigellosis due to Shigella sonnei | | |
| A03.8 | | | Other shigellosis | | |
| A03.9 | | | Shingellosis, unspecified | | |
| A07.1 | | | Giardiasis | | |
| A00.0 | | | Cholera due to Vibrio cholerae 01, biovar cholerae | | |
| A00.1 | | | Cholera due to Vibrio cholerae 01, biovar eltor | | |
| A00.9 | | | Cholera, unspecified | | |
| A01.0 | | | Typhoid fever | | |
| A01.1 | | | Paratyphoid fever A | | |
| A01.2 | | | Paratyphoid fever B | | |
| A01.3 | | | Paratyphoid fever C | | |
| A01.4 | | | Paratyphoid fever, unspecified | | |
| A09.0 | | | Other and unspecified gastroenteritis and colitis of infectious origin | | |
| A09.9 | | | Gastroenteritis and colitis of unspecified origin | | |
| A04.0 | | | Enteropathogenic Escherichia coli infection | | |
| A04.1 | | | Enterotoxigenic Escherichia coli infection | | |
| A04.2 | | | Enteroinvasive Escherichia coli infection | | |
| A04.3 | | | Enterohaemorrhagic Escherichia coli infection | | |
| A04.4 | | | Other intestinal Escherichia coli infections | | |
| A04.5 | | | Campylobacter enteritis | | |
| A04.6 | | | Enteritis due to Yersinia enterocolitica | | |
| A04.7 | | | Enterocolitis due to Clostridium difficile | | |
| A04.8 | | | Other specified bacterial intestinal infections | | |
| A04.9 | | | Bacterial intestinal infection, unspecified | | |
| B98.0 | | | Helicobacter pylori [H.pylori] as the cause of diseases classified to other chapters | | |
|  | | | **Meningitis** | | |
| A39.0 | | | Meningococcal meningitis | | |
| A39.1 | | | Waterhouse-Friderichsen syndrome | | |
| A39.2 | | | Acute meningococcaemia | | |
| A39.3 | | | Chronic meningococcaemia | | |
| A39.4 | | | Meningococcaemia, unspecified | | |
| A39.5 | | | Meningococcal heart disease | | |
| A39.8 | | | Other meningococcal infections | | |
| A39.9 | | | Meningococcal infection, unspecified | | |
|  | | | **Sepsis** | | |
| A40.0 | | | Sepsis due to streptococcus, group A | | |
| A40.1 | | | Sepsis due to streptococcus, group B | | |
| A40.2 | | | Sepsis due to streptococcus, group D | | |
| A40.3 | | | Sepsis due to Streptococcus pneumoniae | | |
| A40.8 | | | Other streptococcal sepsis | | |
| A40.9 | | | Streptococcal sepsis, unspecified | | |
| A41.0 | | | Sepsis due to Staphylococcus aureus | | |
| A41.1 | | | Sepsis due to other specified staphylococcus | | |
| A41.2 | | | Sepsis due to unspecified staphylococcus | | |
| A41.3 | | | Sepsis due to Haemophilus influenzae | | |
| A41.4 | | | Sepsis due to anaerobes | | |
| A41.5 | | | Sepsis due to other Gram-negative organisms | | |
| A41.8 | | | Other specified sepsis | | |
| A41.9 | | | Sepsis, unspecified | | |
| T81.44% | | | Sepsis following procedure | | |
|  | | | **Genitourinary infections** | | |
| N10 | | | Acute tubulo-interstitial nephritis | | |
| N11.0 | | | Nonobstructive reflux-associated chronic pyelonephritis | | |
| N11.1 | | | Chronic obstructive pyelonephritis | | |
| N11.8 | | | Other chronic tubulo-interstitial nephritis | | |
| N11.9 | | | Chronic tubulo-interstitial nephritis, unspecified | | |
| N12 | | | Tubulo-interstitial nephritis, not specified as acute or chronic | | |
| N16.0 | | | Renal tubulo-interstitial disorders in infectious and parasitic diseases classified elsewhere | | |
| N30.0 | | | Acute cystitis | | |
| N30.1 | | | Interstitial cystitis (chronic) | | |
| N30.2 | | | Other chronic cystitis | | |
| N30.3 | | | Trigonitis | | |
| N30.4 | | | Irradiation cystitis | | |
| N30.8 | | | Other cystitis | | |
| N30.9 | | | Cystitis, unspecified | | |
| N39.0 | | | Urinary tract infection, site not specified | | |
|  | | | **Joint infection** | | |
| M00% | | | Pyogenic arthritis | | |
| M01% | | | Direct infections of joint in infectious and parasitic diseases classified elsewhere | | |
| M02% | | | Postinfective and reactive arthropathies | | |
|  | | | **Skin infection** | | |
| B95.6% | | | Staphylococcus aureus as the cause of diseases classified elsewhere | | |
| L03.0% | | | Cellulitis and acute lymphangitis of finger and toe | | |
| L03.1% | | | Cellulitis and acute lymphangitis of other parts of limb | | |
| L03.2% | | | Cellulitis and acute lymphangitis of face and neck | | |
| L03.3% | | | Cellulitis and acute lymphangitis of trunk | | |
| L03.8% | | | Cellulitis and acute lymphanitis of other sites | | |
| L03.9% | | | Cellulitis and acute lymphanitis, unspecified | | |
| L08.8% | | | Other specified local infection of the skin and subcutaneous tissue | | |
| L08.9 | | | Local infection of the skin and subcutaneous tissue, unspec | | |
|  | **Kidney Disease** | | |  |  |
| N29 | Other disorders of kidney and ureter in diseases EC | | |  |  |
| N27 | Small kidney of unknown cause | | |  |  |
| N28 | Other disorders of kidney and ureter NEC | | |  |  |
| N10 | Acute tubulo-interstitial nephritis | | |  |  |
| N05 | Unspecified nephritic syndrome | | |  |  |
| N06 | Isolated proteinuria with specified morphological lesion | | |  |  |
| N20 | Calculus of kidney and ureter | | |  |  |
| N21 | Calculus of lower urinary tract | | |  |  |
| N22 | Calculus of urinary tract in diseases classified elsewhere | | |  |  |
| N25 | Disorders resulting from impaired renal tubular function | | |  |  |
| N17 | Acute renal failure | |  |  |  |
| N13 | Obstructive and reflux uropathy | | |  |  |
| N01 | Rapidly progressive nephritic syndrome | | |  |  |
| N26 | Unspecified contracted kidney | | |  |  |
| N15 | Other renal tubulo-interstitial diseases | | |  |  |
| N19 | Unspecified renal failure | | |  |  |
| N07 | Hereditary nephropathy, not elsewhere classified | | |  |  |
| N02 | Recurrent and persistent haematuria | | |  |  |
| N03 | Chronic nephritic syndrome | | |  |  |
| N04 | Nephrotic syndrome | | |  |  |
| N16 | Renal tubulo-interstitial disorders in diseases EC | | |  |  |
| N08 | Glomerular disorders in diseases classified elsewhere | | |  |  |
| N11 | Chronic tubulo-interstitial nephritis | | |  |  |
| N12 | Tubulo-interstitial nephritis not spec as acute or chronic | | |  |  |
| N00 | Acute nephritic syndrome | | |  |  |
| N23 | Unspecified renal colic | | |  |  |
| N14 | Drug/heavy-metal-induced tubulo-interstitial and tub conds | | |  |  |
| N18 | Chronic renal failure | | |  |  |
