# Supplementary material for: Real-world use of an etanercept biosimilar including selective versus automatic substitution in inflammatory arthritis patients: a UK-based electronic health records study
Source: Rheumatol Adv Pract. 2022 Jul 27;6(2):rkac056. doi: 10.1093/rap/rkac056 (PMC9336562; doi:10.1093/rap/rkac056)
Supplement: rkac056_Supplementary_Data [file rkac056_supplementary_data.zip › Supplementary_Table_S4.docx]

**Supplementary Table S4: Orthopaedic surgery as indicated by OPCS-4 intervention and procedure codes from secondary care datasets**

| **OPCS-4 Code** | **OPCS-4 Term** |
| --- | --- |
| **W37** | **Monk total replacement of hip joint using cement** |
| W370 | Convrs from cement tot hip rep Conversion from cemented total hip replacement |
| W371 | Primary cemented tot hip repl Primary cemented total hip replacement |
| W372 | Conv to cemented tot hip repl Conversion to cemented total hip replacement |
| W373 | Revsion cemented total hip rep Revision cemented total hip replacement |
| W378 | Tot prosth repl hip + cem OS Total prosthetic replacement of hip joint using cement OS |
| W379 | Tot prosth repl hip + cem NOS Total prosthetic replacement of hip joint using cement NOS |
| **W38** | **Lord total hip replace no cem Lord total replacement of hip joint not using cement** |
| W380 | Conv from uncement tot hip rep Conversion from uncemented total hip replacement |
| W381 | Pry uncmnt tot hip replacement Primary uncemented total hip replacement |
| W382 | Cnvrs to uncmnt tot hip rplcmn Conversion to uncemented total hip replacement |
| W383 | Revisn uncemented tot hip rep Revision uncemented total hip replacement |
| W388 | Tot prosth repl hip no cem OS Total prosthetic replacement hip joint not using cement OS |
| W389 | Tot prosth repl hip no cem NOS Total prosthetic replacement hip joint not using cement NOS |
| **W39** | **Other total prosth repl hip Other total prosthetic replacement of hip joint** |
| W390 | Removal prev tot hip repl NEC Removal previous total prosthetic replacement hip joint NEC |
| W391 | Pry hybrid tot hip rplcmnt NEC Primary hybrid total hip replacement NEC |
| W392 | Cnvrs to hbrd tot hip rplc NEC Conversion to hybrid total hip replacement NEC |
| W393 | Revisn hybrid tot hip rep NEC Revision hybrid total hip replacement NEC |
| W394 | Attntn to tot hip rplcmnt NEC Attention to total hip replacement NEC |
| W398 | Total prosthet replace hip OS Other specified total prosthetic replacement of hip joint |
| W399 | Total prosthet replace hip NOS Total prosthetic replacement of hip joint NOS |
| **W40** | **Shiers total knee replacement Shiers total replacement of knee joint using cement** |
| **W40-W87** | **Knee joint operations** |
| W400 | Con from cemented tot knee rep Conversion from cemented total knee replacement |
| W401 | Pry cemented total knee replac Primary cemented total knee replacement |
| W402 | Conv to cemented tot knee repl Conversion to cemented total knee replacement |
| W403 | Revision cemented tot knee rep Revision cemented total knee replacement |
| W408 | Tot prosth repl knee + cem OS Total prosthetic replacement of knee joint using cement OS |
| W409 | Tot prosth repl knee + cem NOS Total prosthetic replacement of knee joint using cement NOS |
| W41 | Arthroplasty knee no cement Arthroplasty of knee joint not using cement |
| W410 | Removal prv uncem tot knee rep Removal previous uncemented total prosthet replacement knee |
| W411 | Primary uncmnt tot knee repl Primary uncemented total knee replacement |
| W412 | Cnvrs to uncmnt tot knee repl Conversion to uncemented total knee replacement |
| W413 | Revision uncmnt tot knee repl Revision uncemented total knee replacement |
| W418 | Tot pros repl knee no cem OS Total prosthetic replacement knee joint not using cement OS |
| W419 | Tot pros repl knee no cem NOS Total prosthetic replacement knee joint not using cement NOS |
| **W42** | **Other arthroplasty knee joint Other arthroplasty of knee joint** |
| W420 | Cnv fr hybrd tot knee repl NEC Conversion from hybrid total knee replacement NEC |
| W421 | Pry hybrid tot knee repl NEC Primary hybrid total knee replacement NEC |
| W422 | Con to hybrid tot knee rep NEC Conversion to hybrid total knee replacement NEC |
| W423 | Revi hybrid tot knee repl NEC Revision of hybrid total knee replacement NEC |
| W424 | Attention to tot knee repl NEC Attention to total knee replacement NEC |
| W428 | Other tot prosth knee repl OS Other total prosthetic replacement of knee joint OS |
| W429 | Other tot prosth knee rep NOS Other total prosthetic replacement of knee joint NOS |
| **W43** | **Prosth cmntd tot shldr rplcmnt Prosthetic cemented total shoulder replacement** |
| **W43-W45** | **Elbow joint operations** |
| **W43-W92** | **Other joint operations** |
| W430 | Cnvr fr cmnt tot shldr rplcmnt Conversion from cemented total shoulder replacement |
| W431 | Pry cmntd tot shldr replacmnt Primary cemented total shoulder replacement |
| W432 | Cnvr to cmntd tot shldr rplcmn Conversion to cemented total shoulder replacement |
| W433 | Rvsn cmntd tot shldr rplcmnt Revision cemented total shoulder replacement |
| W438 | Tot pros repl oth joint+cem OS Total prosthetic replacement of other joint using cement OS |
| W439 | Tot pr repl oth joint+cem NOS Total prosthetic replacement of other joint using cement NOS |
| **W44** | **Prsth uncmnt tot shldr rplcmnt Prosthetic uncemented total shoulder replacement** |
| W440 | Cnvr fr uncmnt tot shldr rplcm Conversion from uncemented total shoulder replacement |
| W441 | Pry uncmnt tot shldr replcmnt Primary uncemented total shoulder replacement |
| W442 | Cnvr to uncmnt tot shldr rplcm Conversion to uncemented total shoulder replacement |
| W443 | Rvsn uncmntd tot shldr rplcmnt Revision uncemented total shoulder replacement |
| W448 | Other joint repl no cement OS Other total prosthet replacem oth joint not using cement OS |
| W449 | Other joint repl no cement NOS Other total prosthet replacem oth joint not using cement NOS |
| **W45** | **Prosth hybrid tot shldr rplcmn Prosthetic hybrid total shoulder replacement** |
| W450 | Cnvr fr hybrid tot shldr rplcm Conversion from hybrid total shoulder replacement |
| W451 | Pry hybrid tot shldr rplcment Primary hybrid total shoulder replacement |
| W452 | Cnvr to hybrd tot shldr rplcmn Conversion to hybrid total shoulder replacement |
| W453 | Rvsn hybrid total shldr rplcmn Revision hybrid total shoulder replacement |
| W454 | Atten tot prosth joint rep NEC Attention to total prosthetic replacement of joint NEC |
| W454/Y037 | Rem prosth joint (no replace) Removal prosthesis from joint (no replacement) |
| W458 | Other tot prosth repl joint OS Other specified other total prosthetic replacement of joint |
| W459 | Other tot prosth rep joint NOS Other total prosthetic replacement of joint NOS |
| **W46** | **Austin-Moore hemiarthropl hip Austin - Moore hemiarthroplasty of hip joint using cement** |
| W460 | Removal prev cem repl hd femur Removal previous cemented prosthetic replacement head femur |
| W461 | Pry cmntd hemiarthroplasty hip Primary cemented hemiarthroplasty of hip |
| W462 | Cnvrs to cmnt hemiarthrpls hip Conversion to cemented hemiarthroplasty of hip |
| W463 | Rvsn cmntd hemiarthroplsty hip Revision cemented hemiarthroplasty of hip |
| W468 | Oth sp prsth cmntd hemiart hip Other specified prosthetic cemented hemiarthroplasty of hip |
| W469 | Prsth cmnt hemiarthrpl hip NOS Prosthetic cemented hemiarthroplasty of hip NOS |
| **W47** | **Prsth uncmnt hemiarthrpl hip Prosthetic uncemented hemiarthroplasty of hip** |
| W470 | Cnvr fr uncmnt hemiarthrpl hip Conversion from uncemented hemiarthroplasty of hip |
| W471 | Pry uncmntd hemiarthropl hip Primary uncemented hemiarthroplasty of hip |
| W472 | Cnvrs to uncmnt hemiarthrp hip Conversion to uncemented hemiarthroplasty of hip |
| W473 | Rvsn uncmnt hemiarthrpl hip Revision uncemented hemiarthroplasty of hip |
| W478 | Other pros uncem hemiarthr hip Other specified prosthetic uncemented hemiarthroplasty hip |
| W479 | Prsth uncmn hemiarthrp hip NOS Prosthetic uncemented hemiarthroplasty of hip NOS |
| **W48** | **Other arthroplasty head femur Other arthroplasty of head of femur** |
| W480 | Cnvr fr prev hemiarthr hip NEC Conversion from previous hemiarthroplasty of hip NEC |
| W481 | Pry prosth hemiarthrpl hip NEC Primary prosthetic hemiarthroplasty of hip NEC |
| W482 | Cnvr to prsth hemiarth hip NEC Conversion to prosthetic hemiarthroplasty of hip NEC |
| W483 | Rvsn prsth hemiarthrpl hip NEC Revision of prosthetic hemiarthroplasty of hip NEC |
| W484 | Atn to prsth hemiarthr hip NEC Attention to prosthetic hemiarthroplasty of hip NEC |
| W488 | Oth sp oth prsth hemiarthr hip Other specified other prosthetic hemiarthroplasty of hip |
| W489 | Oth prosth hemiarthrpl hip NOS Other prosthetic hemiarthroplasty of hip NOS |
| **W49** | **Pros replacem head hum + cemnt Prosthetic replacement of head of humerus using cement** |
| **W49-W51** | **Shoulder joint operations** |
| W490 | Cnvrsn fr cmnt hemiarthr shldr Conversion from cemented hemiarthroplasty of shoulder |
| W491 | Primary cmntd hemiarthr shldr Primary cemented hemiarthroplasty of shoulder |
| W492 | Cnvrs to cmntd hemiarthr shldr Conversion to cemented hemiarthroplasty of shoulder |
| W493 | Rvsn cmntd hemiarthr shldr Revision cemented hemiarthroplasty of shoulder |
| W498 | Oth spec cmntd hemiarthr shldr Other specified cemented hemiarthroplasty of shoulder |
| W499 | Prosth cmnt hemiarth shldr NOS Prosthetic cemented hemiarthroplasty of shoulder NOS |
| **W50** | **Prosth uncmntd hemiarthr shldr Prosthetic uncemented hemiarthroplasty of shoulder** |
| W500 | Cnvr fr uncmnt hemiarth shldr Conversion from uncemented hemiarthroplasty of shoulder |
| W501 | Primary uncmnt hemiarthr shldr Primary uncemented hemiarthroplasty of shoulder |
| W502 | Cnvr to uncmnt hemiarthr shldr Conversion to uncemented hemiarthroplasty of shoulder |
| W503 | Rvsn uncmntd hemiarthr shldr Revision uncemented hemiarthroplasty of shoulder |
| W508 | Oth spec uncmnt hemiarth shldr Other specified uncemented hemiarthroplasty of shoulder |
| W509 | Pros uncmntd hemiart shldr NOS Prosthetic uncemented hemiarthroplasty of shoulderNOS |
| **W51** | **Other prosth repl head humerus Other prosthetic replacement of head of humerus** |
| W510 | Conv from hyb hemrth shoul NEC Conversion from hemiarthroplasty of shoulder NEC |
| W511 | Prim hybr hemiarth should NEC Primary hybrid hemiarthroplasty of shoulder NEC |
| W512 | Conv-hybr hemiarth should NEC Conversion to hybrid hemiarthroplasty of shoulder NEC |
| W513 | Revis hyb hemiarth should NEC Revision hybrid hemiarthroplasty of shoulder NEC |
| W514 | Atten replace head humerus NEC Attention to prosthetic replacement of head of humerus NEC |
| W518 | Other prosth repl head hum OS Other prosthetic replacement of head of humerus OS |
| W519 | Oth prsth replcmnt hd hum NOS Other prosthetic replacement of head of humerus NOS |
| **W52** | **Cmntd unicmprtmntl kn rplcmnt Cemented unicompartmental knee replacement** |
| W520 | Cnvr fr cmnt unicmprt kn rplcm Conversion from cemented unicompartmental knee replacement |
| W521 | Pry cmntd unicmprtmn kn rplcmn Primary cemented unicompartmental knee replacement |
| W522 | Cnvr to cmnt unicmpr kn rplcmn Conversion to cemented unicompartmental knee replacement |
| W523 | Rvsn cmnt unicmprtmn kn rplcmn Revision cemented unicompartmental knee replacement |
| W528 | Prosth repl oth artic + cem OS Prosthetic replacement articulation oth bone using cement OS |
| W529 | Pros repl oth artic + cem NOS Prosthetic replacement articulatn oth bone using cement NOS |
| **W53** | **Uncmnt unicmprtmnt kn rplcmnt Uncemented unicompartmental knee replacement** |
| W530 | Cnvr fr uncmn unicmprt kn rplc Conversion from uncemented unicompartmental knee replacement |
| W531 | Prmy uncmnt unicmprt kn rplcmn Primary uncemented unicompartmental knee replacement |
| W532 | Cnvr to uncmn unicmprt kn rplc Conversion to uncemented unicompartmental knee replacement |
| W533 | Rvsn uncmnt unicmprtm kn rplcm Revision uncemented unicompartmental knee replacement |
| W538 | Prosth repl articul no cem OS Prosthet replacement articulat oth bone not using cement OS |
| W539 | Prosth repl articul no cem NOS Prosthet replacement articulat oth bone not using cement NOS |
| **W54** | **Hybrid unicomprtmnt kn rplcmnt Hybrid unicompartmental knee replacement** |
| W540 | Cnvr fr hybrd unicmprt kn rplc Conversion from hybrid unicompartmental knee replacement |
| W541 | Pry hybrid unicmprtm kn rplcmn Primary hybrid unicompartmental knee replacement |
| W542 | Cnvr to hybrd unicmpr kn rplcm Conversion to hybrid unicompartmental knee replacement |
| W543 | Rvsn hybrd unicmprtmt kn rplcm Revision hybrid unicompartmental knee replacement |
| W544 | Atten repl articul bone NEC Attention to prosthetic replacement of articulation NEC |
| W548 | Other repl artic oth bone OS Other prosthetic replacement of articulation of oth bone OS |
| W549 | Other repl artic oth bone NOS Other prosthetic replacement of articulation of oth bone NOS |
| **W55** | **Prosth interposit arthroplasty Prosthetic interposition arthroplasty** |
| W550 | Con proth intrpstn arthrpls Conversion from previous prosth interposition arthroplasty |
| W551 | Pry proth interpstn arthrplsty Primary prosthetic interposition arthroplasty |
| W552 | Rvsn proth interpstn arthrplsy Revision of prosthetic interposition arthroplasty |
| W553 | Cnvr to prth intrpstn arthrpls Conversion to prosthetic interposition arthroplasty |
| W554 | Atn prth intrpstn arthrpls NEC Attention to prosthetic interposition arthroplasty NEC |
| W558 | Oth sp prth intrpstn arthrplsy Other specified prosthetic interposition arthroplasty |
| W559 | Proth intrpstn arthroplast NOS Prosthetic interposition arthroplasty NOS |
| **W56** | **Other interposit arthroplasty Other interposition arthroplasty** |
| W560 | Con prev intrpstn arthrpls NEC Conversion from previous interposition arthroplasty NEC |
| W561 | Prim interpos arth MTP joint Primary interposit arthroplasty metatarsophalang joint NEC |
| W562 | Pry interpstn arthroplasty NEC Primary interposition arthroplasty NEC |
| W563 | Rvsn of intrpstn arthrplst NEC Revision of interposition arthroplasty NEC |
| W564 | Cnvr to intrpstn arthrplst NEC Conversion to interposition arthroplasty NEC |
| W568 | Oth sp interpstn recnstrctn jt Other specified interposition reconstruction of joint |
| W569 | Interposit reconstr joint NOS Interposition reconstruction of joint NOS |
| **W57** | **Excision arthroplasty** |
| W570 | Cnvr fr prev excsn arthroplsty Conversion from previous excision arthroplasty |
| W571 | Prim exc arthropl 1st MTP join Primary excision arthroplasty 1st metatarsophalangeal joint |
| W572 | Pry excision arthroplasty NEC Primary excision arthroplasty NEC |
| W572/Z843 | Excision arthroplasty of hip |
| W573 | Rvsn of excision arthroplasty Revision of excision arthroplasty |
| W574 | Cnvrsn to excisn arthroplasty Conversion to excision arthroplasty |
| W578 | Oth sp excsn reconstrctn of jt Other specified excision reconstruction of joint |
| W579 | Exc reconstruction joint NOS Excision reconstruction of joint NOS |
| **W58** | **Other arthroplasty** |
| W580 | Conv fr prev resurf arthroplas Conversion from previous resurfacing arthroplasty of joint |
| W581 | Prim resurfacing arthroplasty Primary resurfacing arthroplasty of joint |
| W582 | Rvsn of resurfcng arthroplasty Revision of resurfacing arthroplasty |
| W588 | Reconstruct joint c free flap Reconstruction of joint with free flap |
| W589 | Other reconstruction joint NOS Other reconstruction of joint NOS |
| **W59** | **Fusion of other toe joint** |
| **W59 -W79** | **Foot joint operations** |
| W591 | Pry arthrodesis 1st MTPJ & rep Primary arthrodesis 1st MTPJ & replace lesser MTPJ |
| W592 | Pry arthrodesis 1st MTPJ & exc Primary arthrodesis 1st MTPJ & excision lesser MTPJ |
| W593 | Fusion of first MTP joint Fusion of first metatarsophalangeal joint of toe |
| W594 | Pry arthrdsis IPJ great toe Primary arthrodesis interphalangeal joint of great toe |
| W595 | Pry arthrdsis IPJ oth toe NEC Primary arthrodesis of interphalangeal joint other toe NEC |
| W596 | Revision arthrdsis toe joint Revision arthrodesis of toe joint |
| W598 | Fusion of first MTP joint OS Other specified fusion of first metatarsophalangeal joint |
| W599 | Fusion of other toe joint NOS |
| **W60** | **Fuse joint extraart bone graft Fusion of other joint and extraarticular bone graft** |
| W600 | Cnvrs fr extrartic arthrod NEC Conversion from extraarticular arthrodesis NEC |
| W601 | Pry extraart arthrodsis NEC Primary extraarticular arthrodesis of joint NEC |
| W602 | Rvsn extrartic arthrodesis NEC Revision extraarticular arthrodesis NEC |
| W603 | Conv to extrart arthrodsis NEC Conversion to extraarticular arthrodesis NEC |
| W608 | Fuse joint & extraart graft OS Fusion of joint and extraarticular bone graft OS |
| W609 | Fuse joint & extraart graf NOS Fusion of joint and extraarticular bone graft NOS |
| **W61** | **Other fuse joint & artic graft Fusion of other joint and other articular bone graft** |
| W610 | Cnv fr prev intraart arthrdsis Conversion from previous intraarticular arthrodesis |
| W611 | Prim arthrod & artic graft NEC Primary arthrodesis and articular bone graft NEC |
| W612 | Rev arthrod & artic graft NEC Revision of arthrodesis and articular bone graft NEC |
| W613 | Conv to arthrod artic graf NEC Conversion to arthrodesis and articular bone graft NEC |
| W618 | Fuse joint & artic graft OS Other specified fusion of joint and articular bone graft |
| W619 | Fuse joint & artic graft NOS Fusion of joint and articular bone graft NOS |
| **W62** | **Other primary fusion of joint** |
| W621 | Prim arthrodes & int fixat NEC Primary arthrodesis and internal fixation of joint NEC |
| W622 | Prim arthrodes & ext fixat NEC Primary arthrodesis and external fixation of joint NEC |
| W628 | Other primary arthrodesis OS Other specified other primary fusion of joint |
| W629 | Simple arthrodesis |
| **W63** | **Revisional fusion of joint** |
| W631 | Revis arthrodes int fixat NEC Revision of arthrodesis and internal fixation NEC |
| W632 | Revis arthrodes ext fixat NEC Revision of arthrodesis and external fixation NEC |
| W638 | Revisional fusion of joint OS Other specified revisional fusion of joint |
| W639 | Revision of arthrodesis NEC |
| **W64** | **Conversion to arthrodesis NEC** |
| W640 | Conversion from prev arthrodes Conversion from previous arthrodesis NEC |
| W641 | Conv to arthrod & int fix NEC Conversion to arthrodesis and internal fixation NEC |
| W642 | Conv to arthrod & ext fix NEC Conversion to arthrodesis and external fixation NEC |
| **V22** | **Prim decompress cervical spine Primary decompression operation on cervical spine** |
| V22-V54 | Cerv and thorac spine ops OS Other specified operations on cervical or thoracic spine |
| V221 | Pry ant dcmpr cx spn crd+fsn Primary ant decompression of cervical spinal cord+fusion |
| V222 | Pry ant dcmprs cx spn crd NEC Primary anterior decompression of cervical spinal cord NEC |
| V223 | Prim foraminotomy cerv spine Primary foraminotomy of cervical spine |
| V228 | Prmry post decomprsn cerv cord Primary posterior decompression cervical cord |
| V229 | Prim decomp cervical spine NOS Primary decompression operation on cervical spine NOS |
| **V23** | **Revis decomp cervical spine Revisional decompression operations on cervical spine** |
| V231 | Rev ant dcmpr crv sp crd+fsn Revisional ant decompression op cervicl spinal cord+fusion |
| V232 | Rev ant decomp cerv cord NEC Revisional anterior decompression cervical spinal cord NEC |
| V233 | Revis foraminotomy cerv spine Revisional foraminotomy of cervical spine |
| V238 | Rev post decomprsn cerv cord Revision posterior decompression cervical cord |
| V239 | Rev decomp cervical spine NOS Revisional decompression of cervical spine NOS |
| **V24** | **Decompress thoracic spine NEC Decompression of thoracic spine NEC** |
| V241 | Prim decompr fusion thor spine Primary decompress thoracic spinal cord fusion thorac spine |
| V242 | Prim decompress thor spine NEC Primary decompression of thoracic spine NEC |
| V243 | Rvsnal post decomprs thor dsc Revisional posterior decompression of thoracic disc |
| V248 | Decompression thorac spine OS Other specified decompression of thoracic spine |
| V249 | Prmry post decompr thorac disc Primary posterior decompression of thoracic disc |
| **V25** | **Primary decomp lumbar spine op Primary decompression operations on lumbar spine** |
| V25-V39 | Lumbar spinal cord operations |
| V25-V54 | Lumbar spine operations OS Other specified operations on lumbar spine |
| V251 | Pry extn pst dcmprs lmb sp+fsn Primary extndd post decompression lumbar spine and fusion |
| V252 | Pry extnd pst dcmpr lmb sp NEC Primary extended posterior decompression lumbar spine NEC |
| V253 | Prmy post dcmprsn lmbr spn+fsn Primary posterior decompression lumbar spine and fusion |
| V254 | Prmy post dcmprsn lmbr spine Primary posterior decompression lumbar spine |
| V255 | Prim post decompr LS cord NEC Primary posterior decompression of lumbar spinal cord NEC |
| V256 | Prmy foraminotomy lmbr spine Primary foraminotomy of lumbar spine |
| V258 | Prim decompr op lumb spine OS Primary decompression operation on lumbar spine OS |
| V259 | Prim decompr op lumb spine NOS Primary decompression operation on lumbar spine NOS |
| **V26** | **Revis decompr ops lumbar spine Revisional decompression operations on lumbar spine** |
| V261 | Rev extnd pst dcmpr lmb sp+fsn Revision extndd post decompression lumbar spine and fusion |
| V262 | Rev extnd pst dcmpr lmb sp NEC Revision extended posterior decompression lumbar spine NEC |
| V263 | Rvsn post dcmprsn lmbr spn+fsn Revision posterior decompression lumbar spine and fusion |
| V264 | Rvsn post dcmprsn lmbr spine Revision posterior decompression lumbar spine |
| V265 | Revis post decomp LS cord NEC Revisional posterior decompression of lumbar spinal cord NEC |
| V266 | Rvsnal foraminotomy lmbr spine Revisional foraminotomy of lumbar spine |
| V268 | Revis decompr op lumbar sp OS Revisional decompression operation on lumbar spine OS |
| V269 | Revis decompr op lumb sp NOS Revisional decompression operation on lumbar spine NOS |
| **V27** | **Decompress unspecified spine Decompression operations on unspecified spine** |
| V27-V54 | Oth spine operations,site unsp Other spine operations, site unspecified |
| V271 | Prim decomp sp cord & fuse NEC Primary decompression of spinal cord & fusion spine jnt NEC |
| V272 | Prim decompr spinal cord NEC Primary decompression of spinal cord NEC |
| V273 | Revis decompr spinal cord NEC Revisional decompression of spinal cord NEC |
| V278 | Decompression of spine OS Other specified decompression of spine |
| V279 | Decompression of spine NOS |
| **V29** | **Primary excis cervical IV disc Primary excision of cervical intervertebral disc** |
| **V29-V54** | **Cerv+thoracic spine ops Cervical and thoracic spine operations** |
| V291 | Pry laminec excis cerv IV disc Primary laminectomy excision of cervical intervert disc |
| V292 | 1st hemilaminec cerv IV disc Primary hemilaminectomy excision of cervical IV disc |
| V293 | 1st fenestration cerv IV disc Primary fenestration excision of cervical intervert disc |
| V294 | Badgeley ant fusion cerv spine Badgeley anterior fusion of cervical spine |
| V295 | 1st ant excis cervic disc NEC Primary anterior excision of cervical intervertebr disc NEC |
| V296 | 1st cervical microdiscectomy Primary microdiscectomy of cervical intervertebral disc |
| V298 | Primary excis cervical disc OS Primary excision of cervical intervertebral disc OS |
| V299 | 1st post exc cervical IV disc Primary posterior excision of cervical intervertebral disc |
| **V30** | **Revis cervical disc excis ops Revisional excision of cervical intervertebral disc ops** |
| V301 | Revis laminec exc cerv IV disc Revisional laminectomy excision of cervical intervert disc |
| V302 | Rev hemilaminect exc cerv disc Revisional hemilaminectomy excision cervical intervert disc |
| V303 | Rev fenestration exc cerv disc Revisional fenestration excision of cervical intervert disc |
| V304 | Rvsn ant excsn cerv disc+fusn Revision anterior excision cervical disc and fusion |
| V305 | Revis ant excis cerv disc NEC Revisional anterior excision cervical intervert disc NEC |
| V306 | Revis cervical microdiscectomy Revisional microdiscectomy of cervical intervertebral disc |
| V308 | Revis post excis cerv disc NEC Revisional posterior excision of cervical intervert disc NEC |
| V309 | Revis cervic IV disc excis NOS Revisional excision of cervical intervertebral disc NOS |
| **V31** | **Excision of thoracic disc NEC Excision of thoracic intervertebral disc NEC** |
| V311 | Prmry ant excsn thor disc+fsn Primary anterior excision thoracic disc and fusion |
| V312 | Prim ant/lat exc thor disc NEC Primary anterolateral excision thoracic intervert disc NEC |
| V313 | Prim costotransversect th disc Primary costotransversectomy of thoracic intervertebral disc |
| V318 | Prim excis thoracic disc OS Primary excision of thoracic intervertebral disc OS |
| V319 | Prim excis thoracic disc NOS Primary excision of thoracic intervertebral disc NOS |
| **V32** | **Rvsn excs thr intrvrt dsc NEC Revisional excision of thoracic intervertebral disc NEC** |
| V321 | Rvsn ant excsn thor disc+fsn Revision anterior excision thoracic disc and fusion |
| V322 | Rev AL excis thoracic disc NEC Revisional anterolateral excision thorac intervert disc NEC |
| V323 | Rev costotransversec thor disc Revisional costotransversectomy thoracic intervertebral disc |
| V328 | Rvsn decomprs+fsn thorac spn Revision decompression and fusion thoracic spine |
| V329 | Revis excis thoracic disc NOS Revisional excision thoracic intervertebral disc NOS |
| **V33** | **Primary lumbar discectomy** |
| V331 | Prim laminect exc lumbar disc Primary laminectomy excision of lumbar intervertebral disc |
| V332 | Prim fenestration lumbar disc Primary fenestration of lumbar intervertebral disc |
| V333 | Prmy ant exc lmbr disc+fusion Primary anterior excision of lumbar disc and fusion |
| V334 | Prmy ant exc lmbr disc NEC Primary anterior excision of lumbar disc NEC |
| V335 | Prmy ant exc lmbr dsc+pst fsn Primary anterior excision of lumbar disc and posterior fusn |
| V336 | Pry ant exc lmb dsc+pst instrm Primary ant excision lumbar disc+post instrumentation |
| V337 | Primary lumbar microdiscectomy |
| V338 | Prmy post excision lumbar disc Primary posterior excision of lumbar disc |
| V339 | Primary lumbar discectomy NOS Primary excision of lumbar intervertebral disc NOS |
| **V34** | **Revisional lumbar discectomy** |
| V341 | Revis laminec exc lumbar disc Revisional laminectomy excision of lumbar intervert disc |
| V342 | Revis fenestr exc lumbar disc Revisional fenestration excision of lumbar intervert disc |
| V343 | Rvsn ant exc lmbr disc+fusion Revisional anterior excision of lumbar disc and fusion |
| V344 | Rvsn ant excisn lmbr disc NEC Revisional anterior excision of lumbar disc NEC |
| V345 | Rvs ant excs lmbr dsc+pst fusn Revisional anterior excision of lumbar disc and post fusion |
| V346 | Rev ant excs lmbrdsc+pst instr Revisional anterior excision lumbar disc + post instrument |
| V347 | Rvsnl lmbr microdiscectomy Revisional lumbar microdiscectomy |
| V348 | Rvsnl post excsn lmbr disc Revisional posterior excision of lumbar disc |
| V349 | Revision lumbar discectomy NOS Revisional excision of lumbar intervertebral disc NOS |
| **V35** | **Excis intervertebral disc NEC Excision of intervertebral disc NEC** |
| V351 | Primary excision IV disc NEC Primary excision of intervertebral disc NEC |
| V352 | Revision excision IV disc NEC Revisional excision of intervertebral disc NEC |
| V358 | Percutaneous discectomy |
| V359 | Excision intervertebr disc NOS Excision of intervertebral disc NOS |
| V359/Y081 | Laser discectomy |
| **V37** | **Prim fusion cervical spine jnt Primary fusion of joint of cervical spine** |
| V371 | Brooks fuse atlantoaxial joint Brooks fusion of atlantoaxial joint |
| V372 | Post fusion joint cx spine NEC Posterior fusion of joint of cervical spine NEC |
| V373 | Transoral fuse atlantoax joint Transoral fusion of atlantoaxial joint |
| V374 | Fusion of atlantooccipital jnt Fusion of atlantooccipital joint |
| V378 | Fusion atlantoaxial joint NEC Fusion of atlantoaxial joint NEC |
| V379 | Cervic spine joint fusion NOS Fusion of joint of cervical spine NOS |
| **V38** | **Oth prim fusion joint lumb sp Other primary fusion of joint of lumbar spine** |
| V381 | Prmry post fusion thorac spine Primary posterior fusion of thoracic spine |
| V382 | Prmry post fusn lmbr spine Primary posterior fusion of lumbar spine |
| V383 | Wiltse posterior fusion spine Wiltse posterior fusion of spine |
| V384 | Pry intrtrns fusn lmbr spn NEC Primary intertransverse fusion lumbar spine NEC |
| V388 | Primrposterolat fusn lmbr spn Primary posterolateral fusion lumbar spine |
| V389 | Prim fusion lumbar spine NOS Primary fusion of joint of lumbar spine NOS |
| **V39** | **Other revision lumbar fusion Other revisional fusion of joint of lumbar spine** |
| V391 | Revisnal fusion cerv spine Revisional fusion of cervical spine |
| V392 | Revis post fusion thorac spine Revisional posterior fusion of joint of thoracic spine |
| V393 | Rvsn post fusn intrlam lmbr sp Revision posterior interlaminar fusion of lumbar spine |
| V394 | Revis post lumbar fusion NEC Revisional posterior fusion of joint of lumbar spine NEC |
| V395 | Rvs intrtrnsv fsn lmbr spn NEC Revision intertransverse fusion of lumbar spine NEC |
| V398 | Rvsn posterolat fusn lmbr spne Revision posterolateral fusion lumbar spine |
| V399 | Revision of lumbar fusion NOS |
| **V41** | **Crct spn defrm+instrmntn Correction of spinal deformity and instrumentation** |
| V411 | Knodt spinal distraction rod Posterior attachment Knodt spinal distraction rod to spine |
| V412 | Crct spn defrm+ant instrmntn Correction of spinal deformity and anterior instrumentation |
| V413 | Removal Crctal spn instrmntn Removal correctional spinal instrumentation |
| V418 | Crct spn dfrm+instr+ped fxn sy Crctn spn deform+instrumnt with pedicular fixation system |
| V419 | Instrumental cor def spine NOS Instrumental correction deformity of spine NOS |
| **V42** | **Other corr deformity spine Other correction of deformity of spine** |
| V421 | Excision of rib hump |
| V422 | Epiphysiodesis of spine Epiphysiodesis of spine - deformity correction |
| V423 | Antrolat release spn defrm+gft Anterolateral release of spinal deformity and graft |
| V428 | Other corr deformity spine OS Other specified correction of deformity of spine |
| V429 | Other corr deformity spine NOS Correction of deformity of spine NOS |
| **V43** | **Extirpation spine lesion NEC Extirpation of lesion of spine NEC** |
| V431 | Excis lesion cervical vertebra Excision of lesion of cervical vertebra |
| V432 | Excis lesion thoracic vertebra Excision of lesion of thoracic vertebra |
| V433 | Excis lesion lumbar vertebra Excision of lesion of lumbar vertebra |
| V438 | Gill excis spondylolisthesis Gill excision of spondylolisthesis |
| V439 | Excision lesion of spine NEC Excision of lesion of spine NEC |
| **V44** | **Spine fracture decompression Decompression of fracture of spine** |
| V441 | Complex decompress # spine Complex decompression of fracture of spine |
| V442 | Anterior decompression # spine Anterior decompression of fracture of spine |
| V443 | Posterior decompress # spine Posterior decompression of fracture of spine |
| V448 | Spine fracture decompress OS Other specified decompression of fracture of spine |
| V449 | Spine fracture decompress NOS Decompression of fracture of spine NOS |
| **V45** | **Other spine fracture reduction Other reduction of fracture of spine** |
| V451 | Open reduct exc facet # spine Open reduction of fracture of spine & excis facet of spine |
| V452 | Open reduction # spine NEC Open reduction of fracture of spine NEC |
| V453 | Manipulative reduction # spine Manipulative reduction of fracture of spine |
| V458 | Spinal extension traction # Spinal extension traction for fracture of spine |
| V459 | Other spine fracture reduc NOS Other reduction of fracture of spine NOS |
| **V46** | **Fixation of fracture of spine** |
| V461 | Pry opn red spn #+int fix+plte Primary open reduc spinal fracture+internal fix+plate |
| V462 | Fixat # spine Harrington rod Fixation of fracture of spine using Harrington rod |
| V463 | Pry opn red spn #+int fix+wire Primary open reduc spinal fracture+internal fix+wire |
| V464 | Halo skull traction # spine Halo skull traction for fracture of spine |
| V468 | Pry op rd sp #+int fix+seg wre Primary open reduc spinal #+intern fix+segmental wire system |
| V469 | Fixation spine fracture NOS Fixation of fracture of spine NOS |
| **V52** | **Other intervertebral disc ops Other operations on intervertebral disc** |
| V521 | Enzyme destruct intervert disc Enzyme destruction of intervertebral disc |
| V522 | Destruction of disc NEC Destruction of intervertebral disc NEC |
| V523 | Discography intervert disc Discography of intervertebral disc |
| V524 | Prim ant/lat biops thorac disc Primary anterolateral biopsy of thoracic intervertebral disc |
| V528 | Other intervert disc op OS Other specified operation on intervertebral disc |
| V529 | Other intervert disc op NOS Operation on intervertebral disc NOS |
| **V54** | **Other ops on spine & vertebra Other ops on spine and vertebra** |
| V541 | Transoral excisn odontoid peg Transoral excision of odontoid peg |
| V542 | Graft of bone to spine NEC |
| V543 | Osteotomy of spine NEC |
| V544 | Injection into paraspinal area |
| V548 | Primary thoracic spine op Primary operation on thoracic spine |
| V549 | Primary thoracic spine op NOS Primary operation on thoracic spine NOS |
| **W01** | **Complex reconstruction thumb Complex reconstruction of thumb** |
| **W01-W05** | **Complex reconstruct hand+foot Complex reconstruction operations on hand and foot** |
| **W01-W92** | **Other bone & joint operations Other bone and joint operations** |
| W011 | Microvasc transf toe to thumb Microvascular transfer of toe to thumb |
| W012 | Pollicisation of finger |
| W013 | Thmb rcn usng bne grft+skn flp Thumb reconstruction using bone graft and skin flap |
| W014 | Thmb recon usng bne lngth proc Thumb reconstruction using bone lengthening procedure |
| W015 | Opponensplasty thumb |
| W018 | Free phalangeal transfer thumb Free phalangeal transfer to thumb |
| W019 | Complex reconstruct thumb NOS Complex reconstruction of thumb NOS |
| **W02** | **Other complex reconstr of hand Other complex reconstruction of hand** |
| W021 | Proximal row carpectomy |
| W022 | Metacarpal supp op on carpus Metacarpal support operation on carpus |
| W023 | Multiple jnt reconstr hand NEC Multiple joint reconstruction of hand NEC |
| W024 | Cmplx soft tiss recons hnd NEC Complex soft tissue reconstruction in hand NEC |
| W028 | Reconstruction hand local flap Reconstruction of hand with local flap |
| W029 | Other complex reconst hand NOS Other complex reconstruction of hand NOS |
| **W03** | **Complex reconstr of forefoot Complex reconstruction of forefoot** |
| W031 | Kessel reconstruction forefoot Kessel reconstruction of forefoot |
| W032 | Helal metatarsal osteotomy |
| W033 | Total correction of claw toe |
| W034 | Robert Jones proced great toe Robert Jones procedure great toe |
| W035 | Fusion joints mid & forefoot Localised fusion of joints of midfoot and forefoot |
| W038 | Correction of metatarsus varus |
| W039 | Complex reconstr forefoot NOS Complex reconstruction of forefoot NOS |
| **W04** | **Complex reconstr of hindfoot Complex reconstruction of hindfoot** |
| W041 | Local fusion hindfoot joints Localised fusion of joints of hindfoot |
| W042 | Dunn triple fusion of foot |
| W043 | Goldthwait hindfoot stabilise Goldthwait stabilisation of hindfoot |
| W044 | Muscle strip from os calcis Stripping of muscle from os calcis |
| W045 | Exc lat wedge & fusion os calc Rel medial soft tiss hindfoot & exc lat wedge fusion os calc |
| W048 | Articular fusion subtalar jnt Articular fusion subtalar joint |
| W049 | Complex reconstr hindfoot NOS Complex reconstruction of hindfoot NOS |
| **W05** | **Prosthetic replacement of bone** |
| W051 | Articul prosthet replace bone Articulated prosthetic replacement of bone |
| W058 | Prosthet replacement bone OS Other specified prosthetic replacement of bone |
| W059 | Prosthet replacement bone NOS Prosthetic replacement of bone NOS |
| **W06** | **Excision of entire bone** |
| W06-W36 | Bone operations |
| W061 | Total excision of cervical rib |
| W062 | Total excision of rib NEC |
| W063 | Total excision of patella |
| W064 | Total excision of sesamoid NEC Total excision of sesamoid bone NEC |
| W065 | Talectomy |
| W066 | Total excision of coccyx |
| W068 | Total excision of bone OS Other specified total excision of bone |
| W069 | Ostectomy NEC |
| W069+W059 | Tot excis & prosthet rep bone Total excision of bone and prosthetic replacement for bone |
| **W07** | **Excision of ectopic bone** |
| W071 | Excision of cross union bone Excision of cross union of bone |
| W072 | Excis periartic ectopic bone Excision of periarticular ectopic bone |
| W073 | Excis intramusc ectopic bone Excision of intramuscular ectopic bone |
| W078 | Excision of ectopic bone OS Other specified excision of ectopic bone |
| W079 | Excision of ectopic bone NOS |
| **W08** | **Other excision of bone** |
| W081 | Excision of tuberosity of bone |
| W082 | Excision of overgrowth of bone |
| W083 | Excision of bony excrescence Excision of excrescence of bone |
| W084 | Excision of fragment of bone |
| W085 | Excision metatarsal head NEC Excision of head of metatarsal bone NEC |
| W085+Z814 | Rttr cuff dcomp-opn acrmplsty Rotator cuff decompression - open acromioplasty |
| W088 | Excision of synostosis |
| W089 | Other excision of bone NOS Excision of bone NOS |
| W089+W051 | Excis bone & art pros rep NEC Excision of bone and articulated prosthetic replacement NEC |
| W089+W059 | Excis bone & prosth repl NEC Excision of bone and prosthetic replacement NEC |
| W089+W329 | Excis bone & bone graft HFQ Excision of bone and bone graft however further qualified |
| **W09** | **Extirpation of lesion of bone** |
| W091 | Excision of lesion of bone |
| W092 | Curett bone lesion & graft HFQ Curettage of lesion of bone and graft HFQ |
| W093 | Curettage lesion of bone NEC Curettage of lesion of bone NEC |
| W094 | Destructn lesion of bone NEC Destruction of lesion of bone NEC |
| W098 | Cryoablation of bone lesion |
| W099 | Extirpation bone lesion NOS Extirpation of lesion of bone NOS |
| **W10** | **Open osteoclasis** |
| W101 | Op osteocl ang cor int fix HFQ Open osteoclasis, angular correction & internal fixation HFQ |
| W102 | Op osteocl ang cor ext fix HFQ Open osteoclasis angular correction & external fixation HFQ |
| W103 | Angulatory osteotomy NEC |
| W104 | Open osteoclasis & int fix NEC Open osteoclasis and internal fixation NEC |
| W105 | Open osteoclasis+ext fxtn NEC Open osteoclasis and external fixation NEC |
| W108 | Rotational osteotomy |
| W109 | Open surgical fracture NOS Open surgical fracture of bone NOS |
| **W11** | **Other surgical bone fracture Other surgical fracture of bone** |
| W111 | Closed osteoclasis |
| W118 | Other surg fracture of bone OS Other specified other surgical fracture of bone |
| W119 | Other surg fracture bone NOS Other surgical fracture of bone NOS |
| **W12** | **Angulation periartic osteotomy Angulation periarticular osteotomy** |
| W121 | Bios ang peri ost int fix HFQ Biosseus angulation periarticul osteotomy & int fixation HFQ |
| W122 | Ang peri osteot & int fix NEC Angulation periarticular osteotomy and internal fixation NEC |
| W123 | Bios ang peri ost ext fix HFQ Biosseus angulation periarticul osteotomy & ext fixation HFQ |
| W124 | Ang peri osteot & ext fix NEC Angulation periarticular osteotomy and external fixation NEC |
| W125 | Biosseus ang peri osteot NEC Biosseus angulation periarticular osteotomy NEC |
| W128 | Akin's osteotomy |
| W129 | Angulat periartic osteot NOS Angulation periarticular division of bone NOS |
| **W13** | **Other periarticular osteotomy** |
| W131 | Rotation periarticular osteot Rotation periarticular osteotomy |
| W132 | Displacement osteotomy |
| W133 | Cuneiform osteotomy |
| W138 | Other periarticular osteot OS Other specified other periarticular division of bone |
| W139 | Other periarticular osteot NOS Other periarticular division of bone NOS |
| **W14** | **Diaphyseal division of bone** |
| W141 | Ang diaph osteot & int fix HFQ Angulation diaphyseal osteotomy and internal fixation HFQ |
| W142 | Ang diaph osteot & ext fix HFQ Angulation diaphyseal osteotomy and external fixation HFQ |
| W143 | Angulation diaphys osteot NEC Angulation diaphyseal osteotomy NEC |
| W144 | Rotat diaph ost & int fix HFQ Rotation diaphyseal osteotomy and internal fixation HFQ |
| W145 | Rotat diaph ost & ext fix HFQ Rotation diaphyseal osteotomy and external fixation HFQ |
| W146 | Rotation diaphys osteotomy NEC Rotation diaphyseal osteotomy NEC |
| W148 | Diaphyseal division of bone OS Other specified diaphyseal division of bone |
| W149 | Diaphyseal division bone NOS Diaphyseal division of bone NOS |
| **W15** | **Osteotomy of bone of foot** |
| W151 | Mitchell hallux valgus osteot Mitchell osteotomy for hallux valgus |
| W152 | Golden hallux valgus operation Golden osteotomy base 1st metatarsal bone for hallux valgus |
| W153 | Hallux valgus osteotomy NEC |
| W154 | Osteotomy head metatarsal Osteotomy of head of metatarsal |
| W155 | Osteotomy of midfoot tarsal |
| W158 | Oth spec osteotomy foot bone Other specified osteotomy of bone of foot |
| W159 | Osteotomy of bone of foot NOS |
| **W16** | **Other division of bone** |
| W161 | Multip osteot & int fix HFQ Multiple osteotomy and internal fixation HFQ |
| W162 | Multip osteot & ext fix HFQ Multiple osteotomy and external fixation HFQ |
| W163 | Multiple osteotomy NEC |
| W164 | Intn fixation of osteotomy NEC Internal fixation of osteotomy NEC |
| W165 | Extn fixation of osteotomy NEC External fixation of osteotomy NEC |
| W168 | Other division of bone OS Other specified other division of bone |
| W169 | Osteotomy NEC |
