# Supplementary material for: Real-world use of an etanercept biosimilar including selective versus automatic substitution in inflammatory arthritis patients: a UK-based electronic health records study
Source: Rheumatol Adv Pract. 2022 Jul 27;6(2):rkac056. doi: 10.1093/rap/rkac056 (PMC9336562; doi:10.1093/rap/rkac056)
Supplement: rkac056_Supplementary_Data [file rkac056_supplementary_data.zip › Supplementary_Table_S5.docx]

**Supplementary Table S5: Comorbidities of ETN biosimilar treated patients within the selective or automatic use area.**

|  | Selective SB4  area (n=105) | Automatic SB4  area (n=260) | Difference 95% (CI) |
| --- | --- | --- | --- |
| Hyperlipidemia | 8.6% (9) | 7.7% (20) | 0.9 (-8.3 to 4.7) |
| Hypertension | 27.6% (29) | 29.2% (76) | 1.6 (-9.0 to 11.2) |
| Kidney disease | 12.4% (13) | 6.9% (18) | 5.5 (-0.8 to 13.5) |
| Cardiovascular disease | 4.8% (5) | 7.3% (19) | 2.5 (-3.9 to 7.2) |
| Diabetes | 5.7% (6) | 9.2% (24) | 3.5 (-3.4 to 8.7) |
| Orthopaedic surgery | 19% (20) | 22.7% (59) | 3.6 (-6.1 to 12) |
