# Supplementary material for: Real-world use of an etanercept biosimilar including selective versus automatic substitution in inflammatory arthritis patients: a UK-based electronic health records study
Source: Rheumatol Adv Pract. 2022 Jul 27;6(2):rkac056. doi: 10.1093/rap/rkac056 (PMC9336562; doi:10.1093/rap/rkac056)
Supplement: rkac056_Supplementary_Data [file rkac056_supplementary_data.zip › Supplementary_Table_S7.docx]

**Supplementary Table S7: Characteristics of ETN originator persistent patients compared to ETN biosimilar persistent patients**

|  | ETN originator persistent  n = 497 | ETN biosimilar persistent  n= 321 | Difference (95% CI) |
| --- | --- | --- | --- |
| Female, % (n) | 62.6% (311) | 57.9% (186) | 4.7 (-2.2 to 11.5) |
| Mean BMI, (SD) | 26.8 (6.1) | 26.4 (5.4) | 0.4 (-0.5 to 1.3) |
| Mean age at diagnosis, (SD) | 55.4 (13.9) | 52.2 (13.7) | 3.2 (1.3 to 5.1) |
| Mean disease duration from when commencing ETN originator/ biosimilar, years (SD)^X^ | 26.9 (19) | 25.8 (16.8) | 1.1 (-1.5 to 3.7) |
| Diagnosis of RA, % (n) | 67.2% (334) | 67.9% (218) | 0.7 (-7.2 to 5.9) |
| Diagnosis of PsA, % (n) | 18.1% (90) | 19.3% (62) | 1.2 (-6.8 to 4.2) |
| Diagnosis of AS, % (n) | 15.5% (77) | 15.3% (49) | 0.2 (-5.0 to 5.2) |
| Use Methotrexate, % (n) | 67.8% (337) | 62.3% (200) | 5.5 (-1.2 to 12.2) |
| Use steroids % (n) | 59% (293) | 56.4% (181) | 2.6 (-4.3 to 9.5) |
| Mean previous csDMARDs, (SD) | 1.6 (0.8) | 1.5 (0.6) | 0.1 (-0.4 to 1.2) |
| Mean ETN originator/ETN biosimilar treatment duration, years (SD)† | 6.9 (2.3) | 2.2 (1.1) | 4.7 (4.4 to 5.0) |
| Previous biologics | 10.5% (52) | 32.7% (105) | 22.3 (16.5 to 28.1)* |
| Treated in automatic switching area | 68% (338) | 68.9% (221) | 0.9 (-7.3 to 5.7) |
| *Comorbidities* |  |  |  |
| Hyperlipidemia | 7.8% (39) | 7.5% (24) | 0.4 (-3.6 to 4.0) |
| Hypertension | 37.8% (188) | 28.7% (92) | 9.2 (2.5 to 15.5)* |
| Kidney disease | 10.5% (52) | 4.7% (15) | 5.8 (2.0 to 9.3)* |
| Cardiovascular disease | 9.7% (48) | 6.9% (22) | 2.8 (-1.2 to 6.5) |
| Diabetes | 10.5% (52) | 7.2% (23) | 3.3 (-0.8 to 7.1) |
| Orthopaedic surgery | 31.2% (155) | 14.6% (47) | 16.6 (10.7 to 22)* |
| ***ETN originator/biosimilar treatment outcomes*** | | | |
| Mean DAS28 pre-treatment, (SD)^ | 5 (1.4) | 4.4 (1.1) | 0.6 (-0.4 to 1.2) |
| Mean DAS28 post-treatment, (SD)^ | 3.5 (1.6) | 3.4 (1.7) | 0.1 (-0.7 to 0.9) |
| Mean difference in DAS28 pre- and post-treatment, (SD)^ | 1.5 (1.7) | 1.0 (1.4) | 0.5 (-0.3 to 1.3) |
| Mean GP encounters pre- treatment, (SD) | 59.4 (53.9) | 85.6 (59.4) | 26.2 (1.4 to 42.0)* |
| Mean GP encounters post- treatment, (SD) | 81.4 (51.5) | 61.3 (48.1) | 20.1 (7.0 to 33.2)* |
| Mean difference in encounters to GP pre- and post-treatment, (SD) | 22 (83.2) | 24.3 (85.6) | 2.3 (-25.3 to 20.7) |

** p=<0.05; ^X^ From first inflammatory arthritis mention in primary care records; †Where no end date is present for ETN biosimilar or no additional drugs have been initiated, patients are assumed to have continued to use the treatment.*

*Missing data: DAS-28 ETN originator persistent patients: 48 scores present within one year pre- and post, 86.9% missing; DAS-28 ETN biosimilar persistent patients: 6 scores present pre- and post, 86.4 % missing.*
