# Supplementary material for: Real-world use of an etanercept biosimilar including selective versus automatic substitution in inflammatory arthritis patients: a UK-based electronic health records study
Source: Rheumatol Adv Pract. 2022 Jul 27;6(2):rkac056. doi: 10.1093/rap/rkac056 (PMC9336562; doi:10.1093/rap/rkac056)
Supplement: rkac056_Supplementary_Data [file rkac056_supplementary_data.zip › 21-157 - Supplementary Figures.docx]

**Supplementary Figure S1: Number of patients commencing etanercept originator or biosimilar by year.**

**Supplementary Figure S2: Etanercept originator and biosimilar survival (naïve patients only)**
